# Changes in mRNA/protein expression and signaling pathways in *in vivo* passaged mouse ovarian cancer cells

**DOI:** 10.1371/journal.pone.0197404

**Published:** 2018-06-21

**Authors:** Qingchun Cai, Qipeng Fan, Aaron Buechlein, David Miller, Kenneth P. Nephew, Sheng Liu, Jun Wan, Yan Xu

**Affiliations:** 1 Department of Obstetrics and Gynecology, Indiana University School of Medicine, Indianapolis, Indiana, United States of America; 2 Medical Sciences, Indiana University School of Medicine, Bloomington, Indiana, United States of America; 3 Collaborative Core for Cancer Bioinformatics (C3B), Indiana University Simon Cancer Center, Indianapolis, Indiana, United States of America; 4 Department of Medical and Molecular Genetics, Indiana University School of Medicine, Indianapolis, Indiana, United States of America; University of Alabama at Birmingham, UNITED STATES

## Abstract

The cure rate for late stage epithelial ovarian cancer (EOC) has not significantly improved over several decades. New and more effective targets and treatment modalities are urgently needed. RNA-seq analyses of a syngeneic EOC cell pair, representing more and less aggressive tumor cells *in vivo* were conducted. Bioinformatics analyses of the RNA-seq data and biological signaling and function studies have identified new targets, such as ZIP4 in EOC. Many up-regulated tumor promoting signaling pathways have been identified which are mainly grouped into three cellular activities: 1) cell proliferation and apoptosis resistance; 2) cell skeleton and adhesion changes; and 3) carbohydrate metabolic reprograming. Unexpectedly, lipid metabolism has been the major down-regulated signaling pathway in the more aggressive EOC cells. In addition, we found that hypoxic responsive genes were at the center stage of regulation and detected functional changes were related to cancer stem cell-like activities. Moreover, our genetic, cellular, biochemical, and lipidomic analyses indicated that cells grown in 2D vs. 3D, or attached vs. suspended had dramatic changes. The important clinical implications of peritoneal cavity floating tumor cells are supported by the data proved in this work. Overall, the RNA-seq data provide a landscape of gene expression alterations during tumor progression.

## Introduction

Epithelial ovarian cancer (EOC) represents the most lethal gynecologic malignancy in the United States. In 2017, approximately 22,000 women were estimated to be diagnosed with ovarian cancer and more than 14,000 deaths attributed to the disease were projected to occur. These numbers have not improved over several decades [1–3]. For those women diagnosed with advanced stage high grade serous ovarian cancer (HGSOC), which accounts for about 70% of EOC cases, less than 30% of patients currently survive more than five years after diagnosis with little improvement in overall survival over the past 40 years [2–4]. This poor outcome is mainly attributed to the development of recurrent disease that is often resistant to chemotherapy. Treatment options for recurrent ovarian cancer are currently limited and not curative, warranting the development of novel therapeutic strategies.

While large-scale integrated genomic analyses have been conducted by the Cancer Genome Atlas research network and other organizations [5], the driver genes, functional players, and critical regulators for each step of EOC development remain to be further identified and characterized. It is well known that the tumor microenvironment plays important roles in tumorigenesis [6, 7] and *in vivo* passaged tumor cells usually acquire enhanced tumor progression abilities, such as SKOV3ip1, HEY-A8 or HEY-1B, and PC-3 and LNCaP derived cell lines [8–11]. Hence, comparing the gene expression alterations in these cell lines is one of the approaches to reveal functionally linked genes. However, many of these cell lines and their parental cells are not fully characterized at the gene expression level.

ID8 syngeneic mouse EOC line was obtained through spontaneous transformation of normal ovarian surface epithelial cells from C57BL6 mice by repetitive passage *in vitro* [12]. These tumor-forming cells were not passaged *in vivo*. To determine the critical processes of EOC development, we have developed a highly aggressive EOC cell line (ID8-P1 and ID8-P2) from ID8-P0 (without *in vivo* passage) through *in vivo* passage in immunocompetent and syngeneic C57BL6 mice [13]. The times to tumor formation and mouse morbidity were reduced from ~90 days for ID8-P0 cells to ~30 days in P1 cells [13]. RNA-seq analysis enables a systems-level understanding of gene expression changes underlying the dramatic tumorigenic changes detected.

The next generation of hallmarks of cancer proposed by Hanahan and Weinberg in 2011 [14] includes emerging hallmarks like deregulating cellular energetics (metabolism reprograming). Along with the emphasis in intracellular signaling networks with several functional circuits (proliferation, motility, viability, cytostasis, and differentiation) and tumor microenvironment, our understanding of the cancer orchestra has been depicted and summarized at a new level. Our RNA-seq and functional analyses have focused on these important hallmarks. In particular, we have demonstrated that *in vivo* passaged ID8 in the peritoneal microenvironment promotes resistance to anoikis in ovarian cancer cells by reprogramming SRC/AKT/ERK signaling [13, 15].

A 'Warburg effect' with increased glycolysis has been observed in ID8-P1 vs. -P0 cells [13, 15]. Intense interest in the 'Warburg effect' has been revived by the discovery that hypoxia- inducible factor 1 (HIF1) reprograms pyruvate oxidation to lactic acid conversion [16]. Most, if not all, of solid cancers encounter hypoxic conditions. Cancer cells acquire the ability to survive hypoxic environments and hypoxia itself can activate adaptive cellular responses that contribute to tumor progression [17, 18]. We have shown previously that the human EOC ascites environment is hypoxic [18].

CSC or tumor-initiating cells (TIC) represent a rare population of undifferentiated oncogenic cells responsible for tumor initiation and maintenance. These cells are important in both tumor recurrence and chemoresistance due to their ability to self-renew, modulate/balance the differentiation of tumor cells, and survive in the presence of conventional treatment [19–22]. Although many CSC markers, including CD133, CD117, CD44, CD24, Hoechst 33342-exfluxing side population (SP), and aldehyde dehydrogenase (ALDH) activity are available to purify TIC [23, 24], our understanding of CSC in EOC is far from complete, partially related to the highly heterogeneous nature of the disease. Thus, the identification and further characterization of CSCs in EOC is critical for the development of new therapeutic strategies for more effective treatment of EOC.

RNA-seq data for mRNA expression in two pairs of ID8-P0 and -PW2 (tumor cells isolated from tumors developed in the peritoneal wall after two *in vivo* passages) cells cultured in suspension were obtained using an improved protocol developed in Dr. Nephew’s lab [25]. In this work, bioinformatic analyses were conducted, which allows the large number of data sets generated in recent years to be integrated. Networks and pathways revealed by bioinformatic analyses guided genetic, cellular, and biochemical analyses to validate and identify new targets for EOC. In particular, the expression, signaling mechanisms, and/or functions of several important targets were verified in human tissues and/or human EOC cells. Many of the identified genes/proteins in the RNA-seq data have been previously shown to be up-regulated in human EOC tissues, with potential clinical correlation, suggesting that the findings have therapeutic implications.

## Results

### RNA-seq data generation and analyses

**Fig 1:**
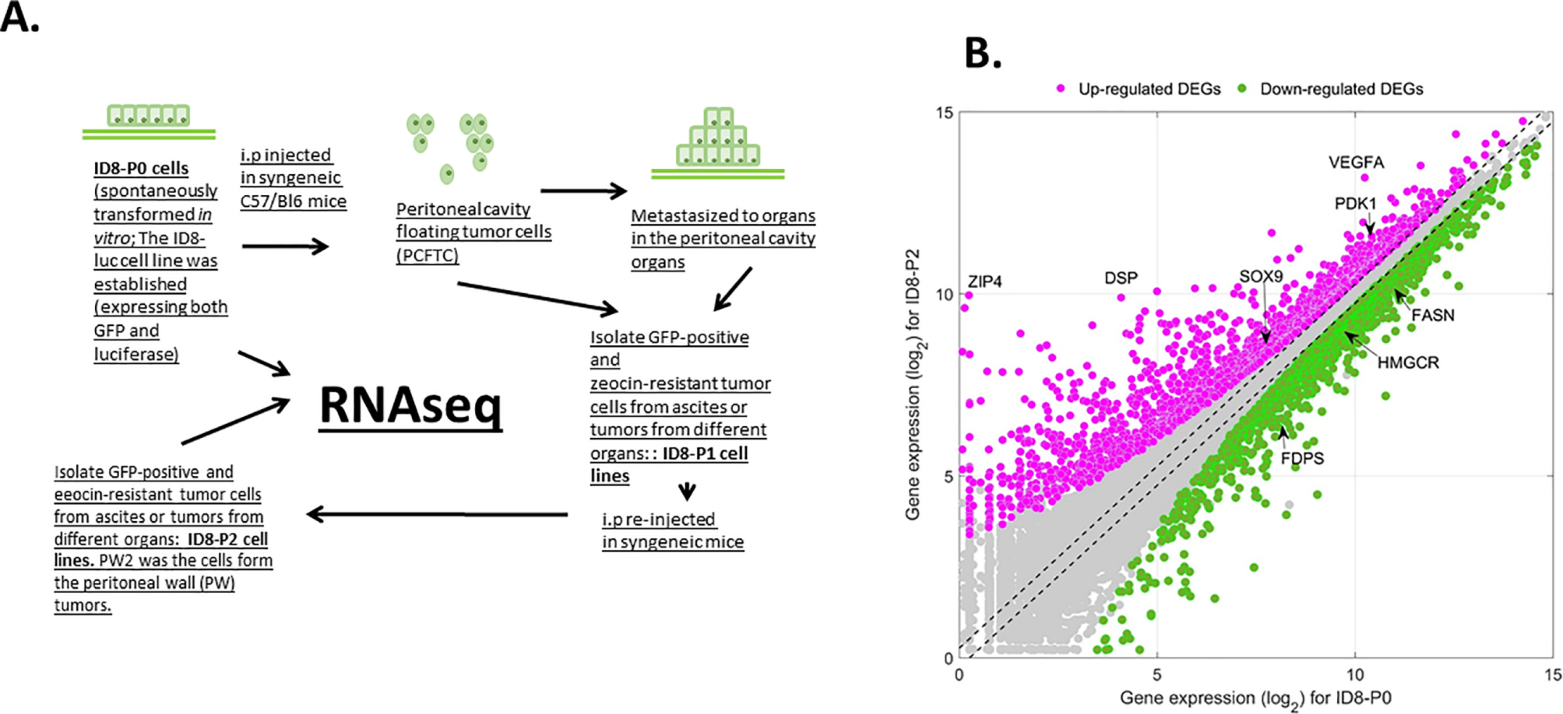
Experimental design and differentially expressed genes (DEG) analyses. **A.** ID8 syngeneic mouse EOC line was obtained through spontaneous transformation of normal ovarian surface epithelial cells from C57BL6 mice by repetitive passage in vitro. The ID8-luc cell line (expressing both GFP and luciferase) was established as described previously. The procedures used to establish ID8-P1 and -P2 cell lines and the cells used for RNA-seq are illustrated in the figure. **B.** Scatter plot of gene expression for ID8-P0 and ID8-P2 cell lines. The red and green dots represent up- and down-regulated DEGs, respectively. Several genes mentioned in the current work are marked in the figure.

RNA-seq data for mRNA expression in two pairs of ID8-P0 and ID8-PW2 [an ID8-P2 cell line derived from tumor cells isolated from tumors in the peritoneal wall (PW) after two *in vivo* passages] cells cultured in suspension condition were generated. The experimental design of the study is shown in **Fig 1A**. While we have developed both ID8-P1 and ID8-P2 (*in vivo* passage 1 and 2) cells, we found that the second *in vivo* passage does not further increase aggressiveness *in vivo* and there are no significant differences in *in vitro* functional analyses, including anoikis resistance and cell proliferation. In addition, the ID8-P1 cells obtained from different organs (including ascites) do not show significant functional differences *in vitro* and have similar tumorigenic potential and metastatic organ preference as we have shown previously [13]. Hence, although the RNA-seq data were generated from the ID8-P2 vs. -P0 cells, most expression and functional assays were conducted and compared between ID8-P1 and -P0 cells in this work.

The duplicate RNA-seq data displayed highly consistent results, evidence by correlational analyses (**S1 Fig**). We confirmed the results using RT-qPCR, Western blot analyses, and/or ELISA assays in ID8 and human EOC cells for more than 40 genes identified. Differential analysis was performed with a published software edgeR [26]. Differentially expressed gene (DEG) was determined if its p-value after false discovery rate (FDR) controlling multiple test correction was less than 0.01 and its fold change (FC) magnitude between ID8-PW2 and -P0 was greater than 2-fold (**Fig 1B**). In this study, we finally identified 1902 up-regulated DEGs in ID8-PW2 and 1466 down-regulated DEGs in ID8PW2 compared to ID8-P0. More than 6,000 genes with detectable expression levels were not significantly changed between the ID8-PW2 and -P0 cells, including those listed as new and revisited housekeeping genes (Chmp2a, Emc7, Psmb2, Psmb4, Snrpd3, and Vps29) identified based on analysis of next-generation sequencing (RNA-seq) data [27], indicating that the expression levels are normalized.

### RNA-seq identified new potential target genes

The top candidate is Slc39A4 or ZIP4, a zinc (Zn) transporter, which was 183-fold upregulated as detected by RNA-seq. We have confirmed that ZIP4 is over-expressed in human EOC tissue and demonstrated its CSC-related activities in human high grade serous ovarian cancer (HGSOC) cells. Interestingly, the oncolipid lysophosphatic acid (LPA) effectively up-regulates ZIP4 expression via the nuclear receptor peroxisome proliferator-activated receptor gamma (PPARγ) [28]. Several putative PPRE elements [29], containing the AGGTCA sequence are detected in the 10 kb ZIP4 promoter region.

In addition to ZIP4, we found that several other zinc or metal ion regulating proteins were up-regulated. In fact, the molecular function of GO:0008270~zinc ion binding identified 136 genes upregulated in ID8-PW2 vs. P0 cells (**S1 Table**). Enpp1, Enpp2 (ATX, the major LPA producing enzyme), Brca1, Mt1, Mt2, and Pxn are among the list. We have confirmed the over-expression of Mt1/2, as well as several additional genes (see below).

**Fig 2:**
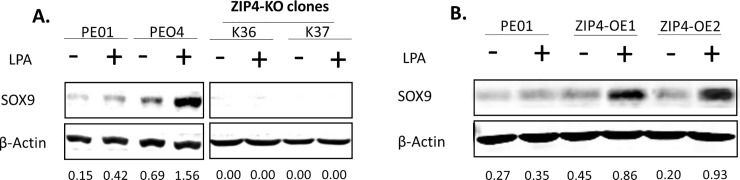
ZIP4 expression regulated SOX9 expression. **A.** In PE04-ZIP4-knockout cell lines (K36 and K37 [28]), SOX9 expression was blocked. **B.** In PE01-ZIP4-overexpression clones, LPA-induced up-regulation of SOX9.

SOX9 is another gene detected to be up-regulated in ID8-P1 vs. ID8-P0 cells (2.6 fold, *P* = 1 x 10^-26^). We have recently determined SOX9’s potential oncogenic activity and regulatory mechanisms controlling SOX9 protein expression in EOC cells (ID8 and HGSOC cells) [30]. SOX9 is involved in cellular activities related to CSC, including anoikis-resistance, regulation CSC marker CD44, and spheroid-formation [30]. Interestingly, ZIP4 was an up-stream regulator of SOX9, since we found that when ZIP4 was knocked out by the CRISPR/Cas9 system in PE04 cells or ZIP4 overexpression (OE) in PE01 cells as shown previously [28], SOX9 expression was completely blocked or upregulated by LPA in a ZIP4-dependent manner, respectively (**Fig 2**).

### Identification of major signaling pathways involved in cancer hallmarks

**Fig 3:**
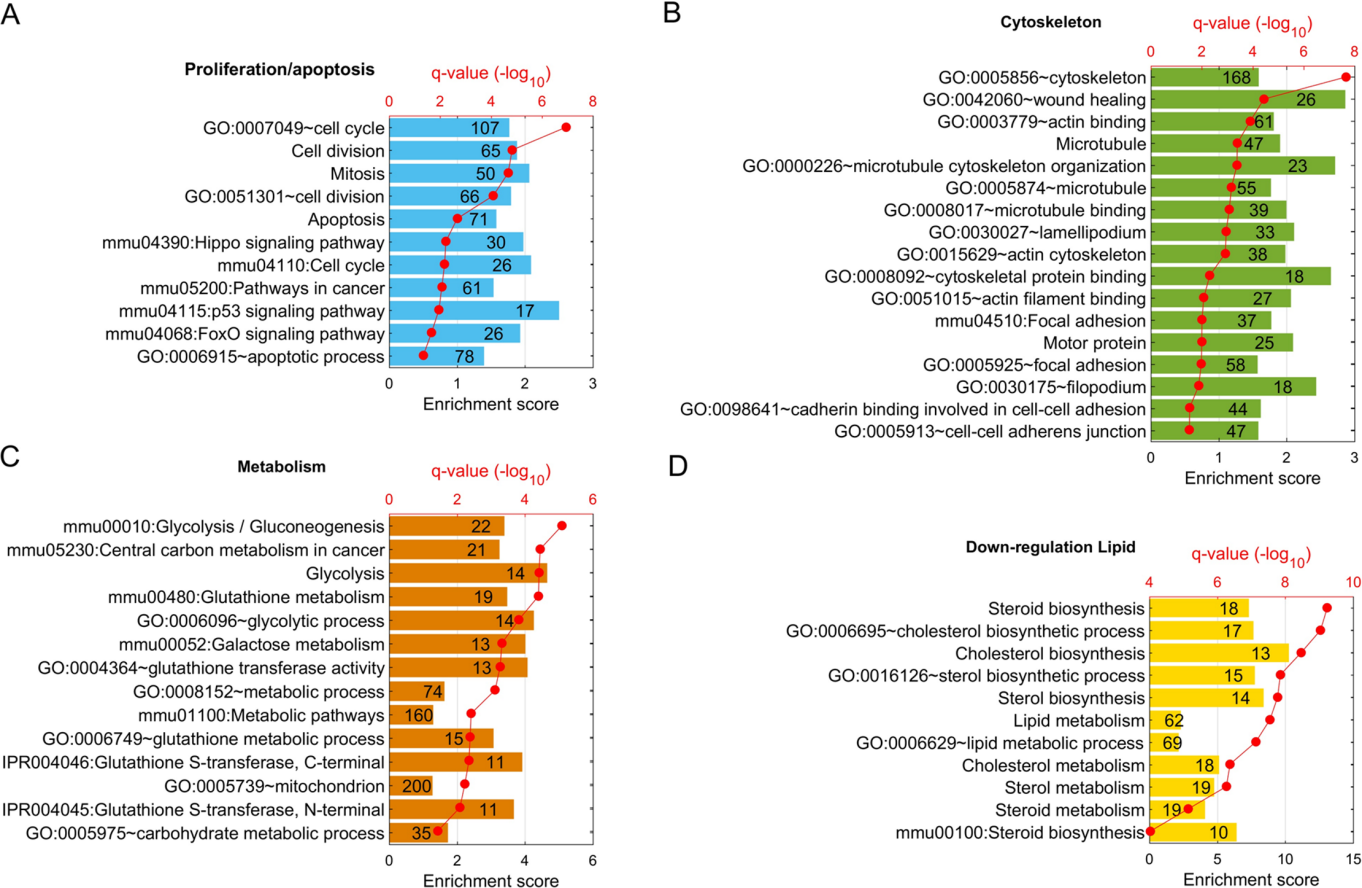
Altered functions and pathways in ID8-PW2 vs. -P0 cells. GO functions and pathways significantly enriched in altered genes are grouped based on four major cellular processes. **A.** Cell proliferation and apoptosis-resistance. **B.** Cytoskelton/cell adhesion. C. Metabolism (glucose and amino acids). D. Lipid metabolism. Q values (p-values corrected by FDR multiple test adjustment) for corresponding functions and pathways are shown as red dots.

Functional enrichment analyses on DEGs were completed by using an online tool, DAVID (https://david.ncifcrf.gov,v6.8). A number of gene ontology (GO) functions, KEGG pathways (KEGG is a collection of databases dealing with genomes, biological pathways, diseases, drugs, and chemical substances), and other functional annotations were recognized as significantly over-represented in DEGs up- or down-regulated in ID8-PW2 compared to ID8-P0. These signaling pathways are involved in several major cancer hallmarks [14], including pathways controlling cell proliferation; resisting cell death; enabling replicative immortality [such as GO:0007049~cell cycle, GO:0051301~cell division, mmu04110:Cell cycle, and GO:0006915~apoptotic process (anti-apoptosis gene upregulation); **Fig 3A**]; and activating cell skeleton changes, which may result in invasion and metastasis or altered cell-cell and cell matrix adhesion (such as GO:0005856~cytoskeleton, GO:0042060~wound healing, GO: 0030027 ~lamellipodium, and mmu04510:Focal adhesion; **Fig 3B**). In addition, reprogramming of carbohydrate metabolism, amino acid, drug, xenobiotics, and redox metabolism was prominently involved (such as mmu00010:Glycolysis/Gluconeogenesis, mmu00480:Glutathione metabolism, GO:0006096~glycolytic process, GO:0004364~glutathione transferase activity, GO:0008152~metabolic process; **Fig 3C**). Unexpectedly, biosynthesis and metabolism of cholesterol, sterol, and lipids were down- regulated pathways (**Fig 3D**). The genes listed in these pathways and their FCs are shown in **S2 Table**.

### Cell survival under stress was critical for EOC progression

The bioinformatic data analyses related to cell survival/apoptosis (**Fig 3A** and **S2 Table**) are consistent and supported by our published data [16, 29, 31] and new data presented here. In our original paper describing ID8-P0 and ID8-P1 cells, we found that enhanced anoikis resistance is a key cellular process associated with greater aggressiveness and tumorigenicity *in vivo* [13]. A higher rate of metabolism and autophagy are also associated with increased anoikis resistance [13].

### Genes related to cell-cell adhesion and EMT/MET were differentially regulated after *in vivo* passaging

As shown in **Fig 3B** and **S2 Table**, many genes involved in cell skeleton, cell-cell, and cell matrix adhesion were up-regulated. Among the top six genes up-regulated in ID8-P1 cells, two genes encode important desmosome components, Dsp and Pkp1. Desmoplakin (Dsp, 55.4-fold up; *P* = 1.2 x 10^-196^) is an obligate component of functional desmosomes that anchors intermediate filaments to desmosomal plaques [31]. Plakophilin 1 (Pkp1, 493-fold up; *P* = 2.1 x 10^-167^) encodes a member of the arm-repeat (armadillo) and plakophilin (PKP) gene families. PKPs contain numerous armadillo repeats, localize to cell desmosomes and nuclei, and participate in linking cadherins to intermediate filaments in the cytoskeleton [32].

**Fig 4:**
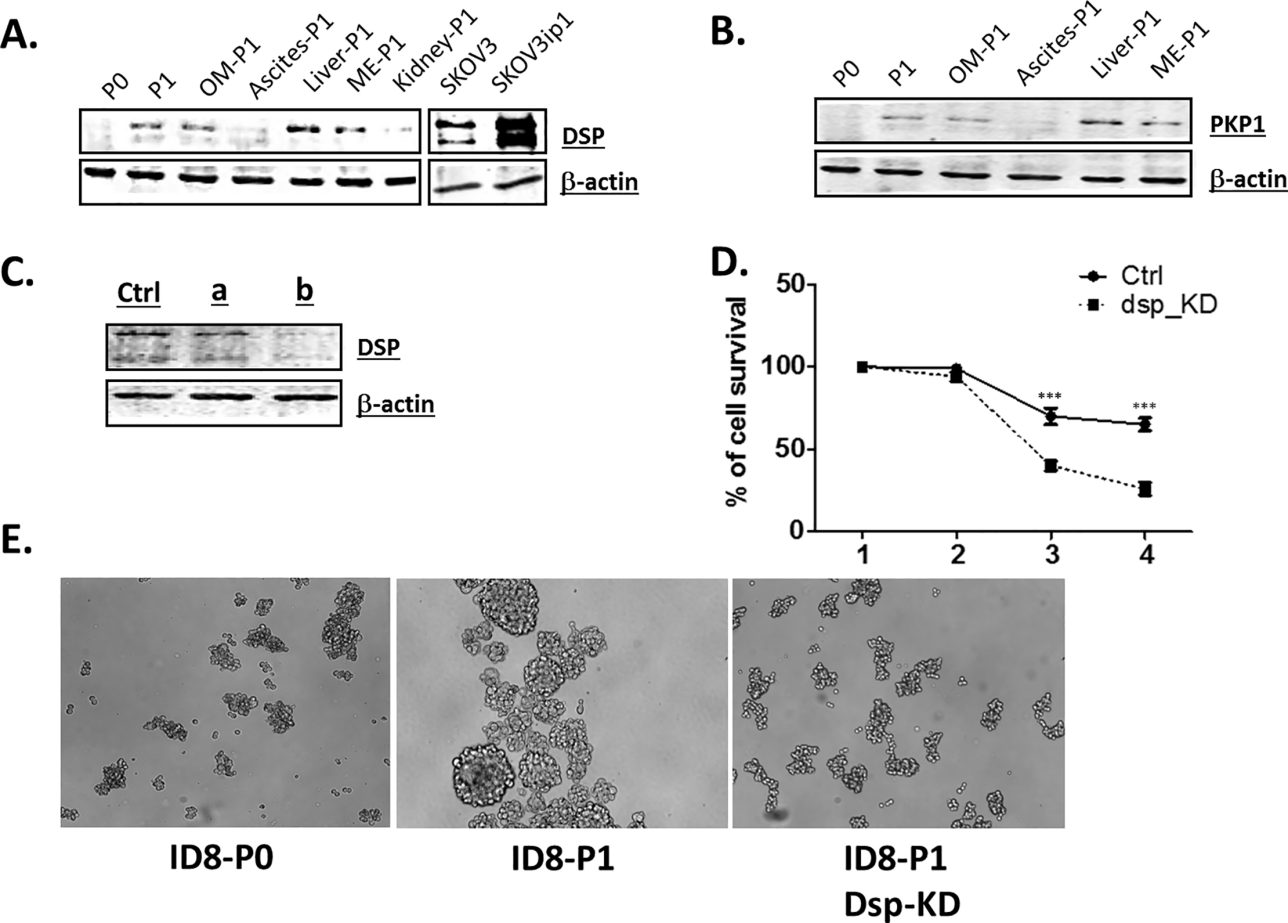
DSP and PKP1 were up-regulated in *in vivo* passaged EOC cells. **A.** DSP protein was upregulated in different P1 cell lines vs. P0 cells: PW, Peritoneal wall metastases-derived P1 cells; OM, omentum metastases-derived P1 cells; Liver, liver metastases-derived P1 cells; Kidney, kidney metastases-derived P1 cells; ME, mesentery metastases-derived P1 cells; P2, PW-P1 cells *in vivo* passage one more time; SKOV3ip1, i.p injected SKOV3 cells. **B.** PKP1 protein was upregulated in different P1 cell lines vs. P0 cells. **C.** Knock-down (KD) DSP clones (a and b) in PW-P1 cells by shRNA. The first lane were from PW-P1 transfected with control (ctrl) shRNA. **D.** Representative data showing that DSP was involved in anoikis-resistance. Cell survival was analyzed in cell suspension conditions in the presence of 2% FBS. **E.** The cell aggregation in P1 cells was inhibited by KD Dsp. Cells were cultured under suspension conditions for 48 hr.

We confirmed Dsp and Pkp1 up-regulation in different ID8-P1 cell lines, as well as in the human EOC SKOV3ip1 vs. SKOV3 cell pairs (**Fig 4A and 4B**). DSP’s functional involvement in EOC in anoikis-resistance was shown in shRNA-mediated DSP-knocked down (KD) stable cell lines (**Fig 4C and 4D**). We detected two bands for Dsp (**Fig 4A**). Our KD experiments using shRNA (**Fig 4C**) showed that both of the bands were affected, suggesting that they both belong to Dsp. Whether these two bands represent different post-translational modification(s) is unknown and requires further investigation. In addition, P1 cells aggregated more and P0 cells tended to be single cells when they were cultured in suspension. Dsp-KD dissociated cell aggragates/spheroids (**Fig 4E**), suggesting that desmosomes play an important role in spheroid formation, which may be directly related to anoikis-resistance and the CSC-like activities.

Compared to other solid adenocarcinomas, which metastasize hematogenously, metastasis of EOC occurs primarily via peritoneal cavity dissemination, characterized by exfoliation of cells from the primary tumor, avoidance of detachment-induced cell death (anoikis), movement throughout the peritoneal cavity as individual cells and multi-cellular aggregates, adhesion to and disruption of the mesothelial lining of the peritoneum, and submesothelial matrix anchoring and proliferation to generate widely disseminated metastases [33]. These steps are accompanied by phenotypic plasticity, enabling dynamic mesenchymal-to-epithelial (MET) and epithelial-to-mesenchymal (EMT) transitions [33]. The abnormal expression of several additional cell-cell adhesion molecules is reflected by their expression changes: Ncam1 (6.2 x, *P* = 2.76x10^-73^); Cdh1) (E-cadherin, 1.6 x, *P* = 0.02); Cdh2 (N-cadherin, not changed *P* = 0.79); Cdh3) (P-cadherin, 3.1 x, *P* = 8.9 x 10^-18^); Epcam (4.9 x, *P* = 1.3 x 10^-8^), Cdh23 (Otocadherin, 0.41 x, *P* = 1.9 x 10^-6^), Vim (Vimentin, 1.4 x, *P* = 2.0 x 10^-7^), Tjp1 (ZO-1, 1.2 x, *P* = 0.004), Zeb1 (no change, *P* = 0.24), Zeb2 (1.3x, *P* = 0.004), Tgfb1 (1.9 x, *P* = 3.3x10^-10^), Tgfb2 (0.41 x, *P* = 2.0x10^-11^) and Ctnnb1 (β-catenin, 1.6 x, *P* = 5.0 x 10^-14^). These data indicate that a more complex and maybe more dynamic EMT/MET occurs in EOC.

### Genes related to cell-matrix adhesion and cell skeleton/focal adhesion were differentially regulated after *in vivo* passaging

The mmu04510:Focal adhesion and the GO:0005925~focal adhesion pathways identified 37 and 58 genes up-regulated in P1 vs. P0 cells, respectively (**Fig 3B** and **S2 Table**). These lists include several integrin genes, including several integins (Itgb5, Itga11, Itga5), several extra cellular matrix proteins [Fn1 (fibronectin 1), Col3a1, Col6a3], and other well know cell-cell and cell-matrix adhesion proteins, as well as cell membrane receptors [such as Tns1, CD44, Pxn (paxilin), Anxa6 (annexin A6), Adam17, Ctnnb1 (β-catenin), Dab2 (Disabled-2), Pak1 (RAC1 Activated Kinase 1), Cttn (cortactin), Fzd1 (frizzled class receptor 1), Palld (Palladin), Rap1b, Pik3cd, Mapk3, Akt3, and Shc1] (**S2 Table**).

### Differential gene expression and function in suspended and attached EOC cells

**Fig 5:**
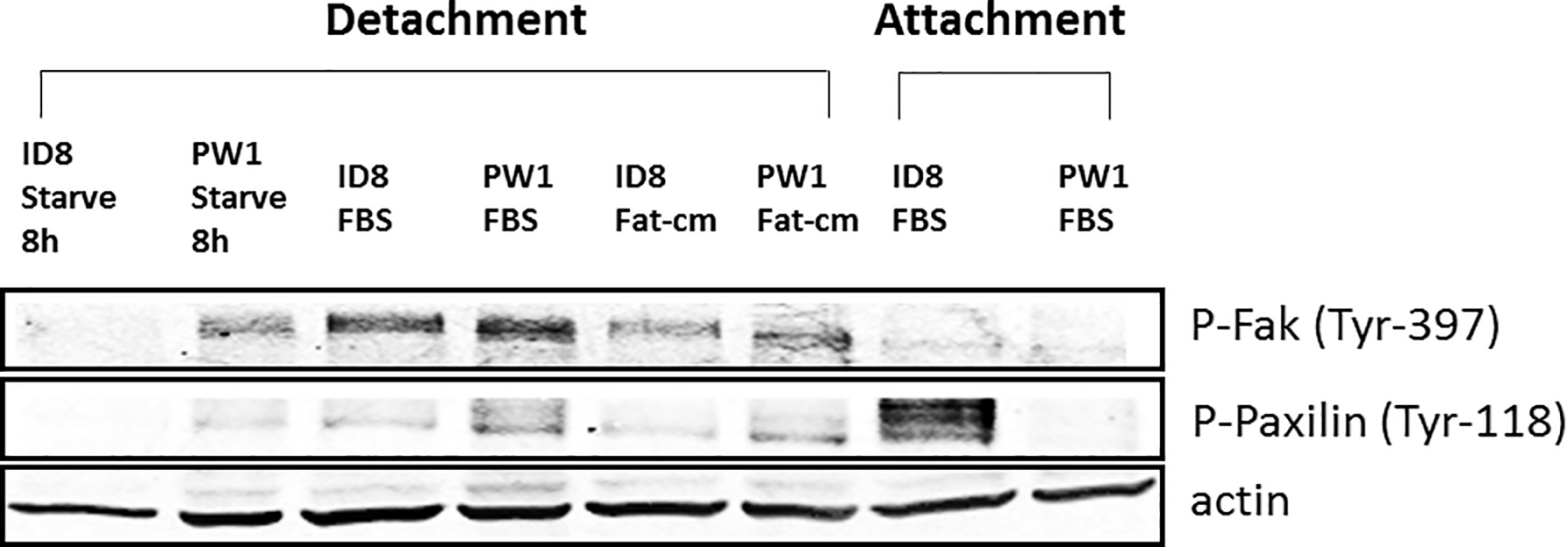
Focal adhesion kinase (FAK) and paxilin (Pxn) activation was regulated by stresses. Phosphorylation of FAK and PXN was mainly upregulated when cells were cultured in suspension under starvation. Western blot analysis showing that ID8-P0, ID8-PW1, or ID8-PE2 cells were cultured in FBS, lipid depleted (charcoal-treated) FBS (Fat-cm), or FBS starved condition in attached or detached conditions.

**Fig 6:**
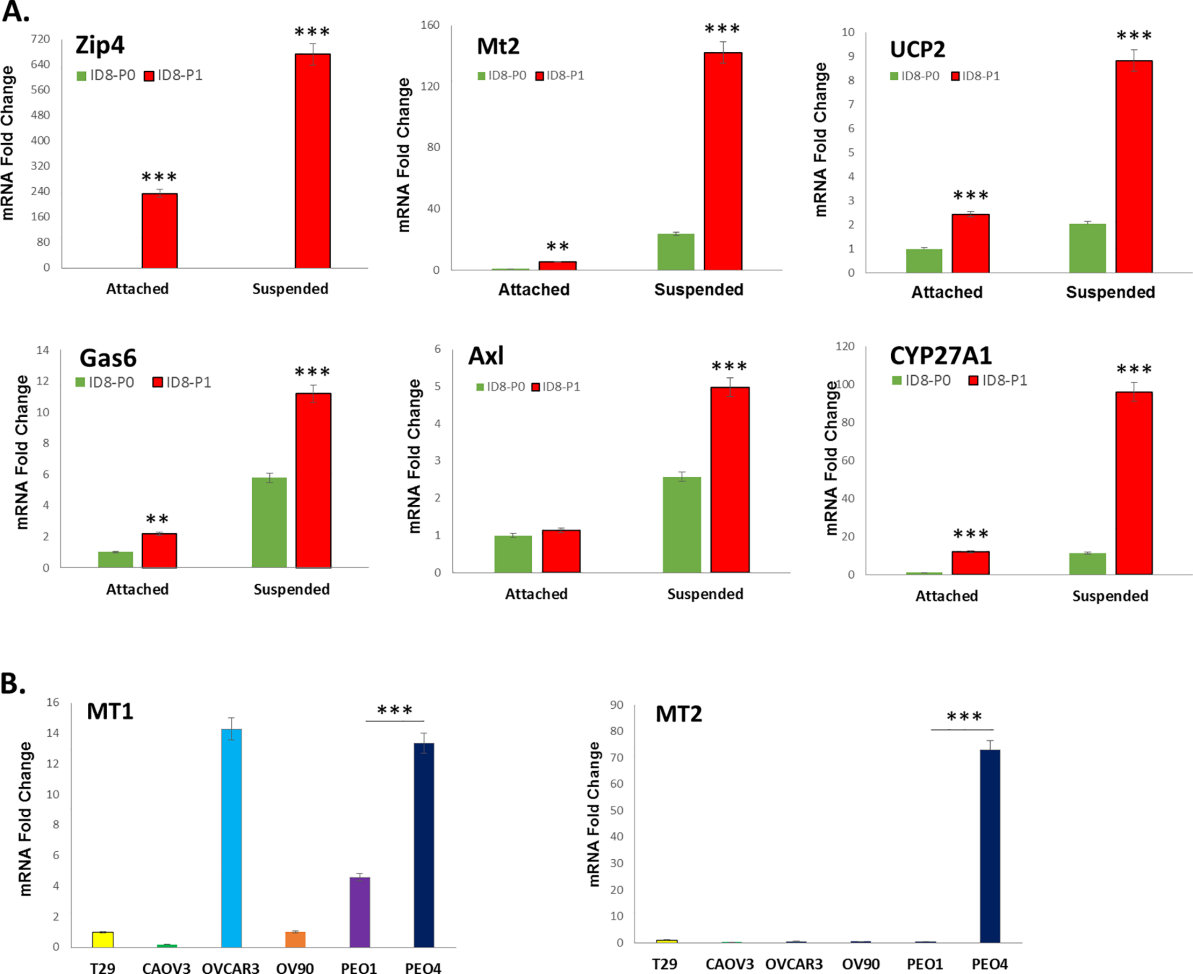
Gene regulation confirmed by Q-PCR in mouse and human EOC cell lines. A-B. Mouse ID8 P0/P1 cells (A) or human EOC cells (B) were seeded into 6-well plates in attached or low-attached plates in suspension, RNAs were extracted with the RNeasy mini kit (Qiagen, Valencia, CA) and reverse transcribed by M-MLV reverse transcriptase. Quantitative real-time PCR was performed on a Light Cycler 480 (Roche, Indianapolis, IN) with a SYBR Green I Master Mix (Roche, Indianapolis, IN). mRNA abundance was normalized to GAPDH.

Interestingly, while the expression levels of focal adhesion kinase (Fak, gene name Ptk2, 1.2 x, *P* = 0.0084) and paxilin (gene name Pxn, 1.3 x, *P* = 0.00036), were not strongly up-regulated, their phosphorylation was enhanced in ID8-P1 vs -P0 cells when cells were cultured in suspension under FBS starvation condition (**Fig 5**), suggesting activation of these gene responses to stresses, such as cell detachment and nutritional deficiency. We further tested several RNA-seq identified and up-regulated genes in ID8-P1 vs. -P0 cells at the mRNA level using Q-PCR under detached vs. attached culture conditions. As shown in **Fig 6A,** the up-regulation of these genes in ID8-P1 vs. ID8-P0 cells were confirmed and detached cells showed more dramatic up-regulation. MT1 and MT2 genes were also expressed in several human HGSOC cell lines and PE04 cells expressed higher levels of these genes than PE01 cells (**Fig 6B**).

### Glycolysis was involved in the increased survivability in ID8-P1 cells

**Fig 3C** and **S2 Table** show that the metabolic pathways and genes (glycolysis, amino acid, glutamine and carbohydrate metabolism, and mitochondrion) were up-regulated in ID8-PW2 vs. -P0 cells. Cells can be rescued from anoikis by enhanced metabolism, which is regulated by HIF1 and AKT signaling [34]. ID8-P1 cells produce more lactate than ID8-P0 cells in culture medium [13], suggesting a Warburg effect. Interestingly, robust pH-regulating systems are needed for bioenergetic processes to combat the excessive generation of lactic and carbonic acids during glycolysis and metabolic reprogramming [17]. We found that pH pathway is also an up-regulated pathway in ID8-PW2 cells (**S3 Table**).

**Fig 7:**
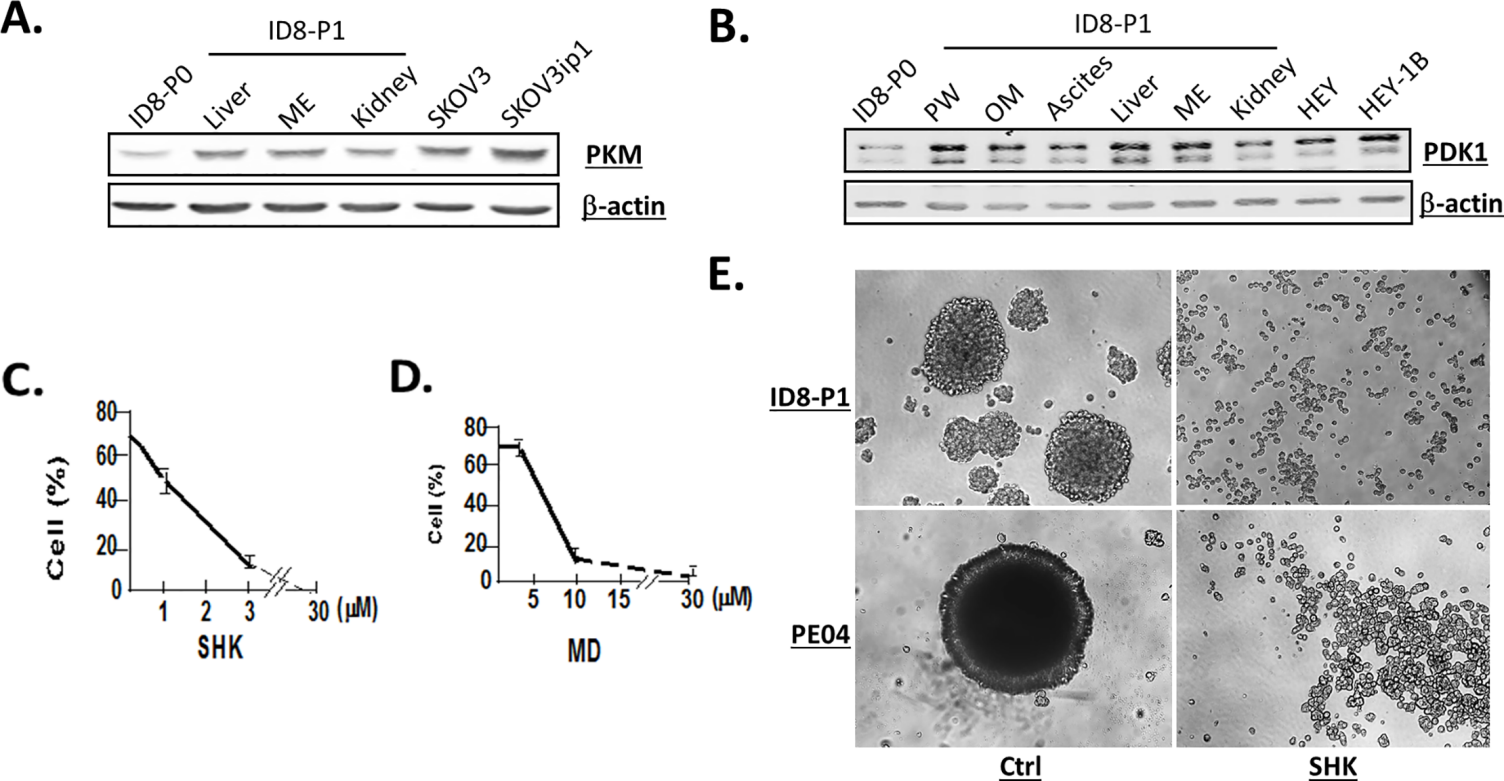
PKM and PDK1 were up-regulated in P1 vs. P0 cells and the PKM2 inhibitors inhibited cellular functions. A and B. PKM2 and PDK1 in P0 and different P1 cells (ME, mesentery, PW, peritoneal wall; OM, omentum) as well as human EOC cells (SKOV3 and HEY). C and D. Pretreatment (1 hr) of PKM inhibitors, shikonin (SHK) and menadione (MD) dose-dependently reduced anoikis- resistance. % of cell survival are shown in the Y-axis. E. ID8-P1 and PE04 formed spheroids was disrupted by SHK (1.25 μM).

Pyruvate dehydrogenase kinase (PDK1) catalyzes phosphorylation of pyruvate dehydrogenase, a key mitochondrial enzyme in the Krebs cycle [16]. Pyruvate kinase muscle (PKM), a rate limiting glycolytic enzyme, catalyzes the final step of glycolysis and is a critical regulator of glucose consumption [35]. Our RNA-seq data show that Pdk1, Pkm, and Glut1 (Slc2a1) genes were 2.2, 1.3, and 1.7-fold up-regulated, respectively, in ID8-P1 vs. -P0 cells, suggesting that these genes are transcriptionally regulated and may be functionally involved in anoikis-resistance. We confirmed that PKM and PDK1 were up-regulated in ID8-P1 vs.–P0 cells (**Fig 7A and 7B**). The nature of the multiple bands of Pdk1 detected is unclear and needs to be further investigated. Shikonin (SHK) and menadione (MD), known PKM2 inhibitors, inhibited anoikis- resistance in ID8-P1 cells (**Fig 7C and 7D**). In addition, we found that SHK reduced cell-cell aggregation and/or spheroid formation in both Murine and human HGSOC cells (**Fig 7E**).

### The hypoxia responsive genes played important roles in more aggressive EOC cells

**Fig 8:**
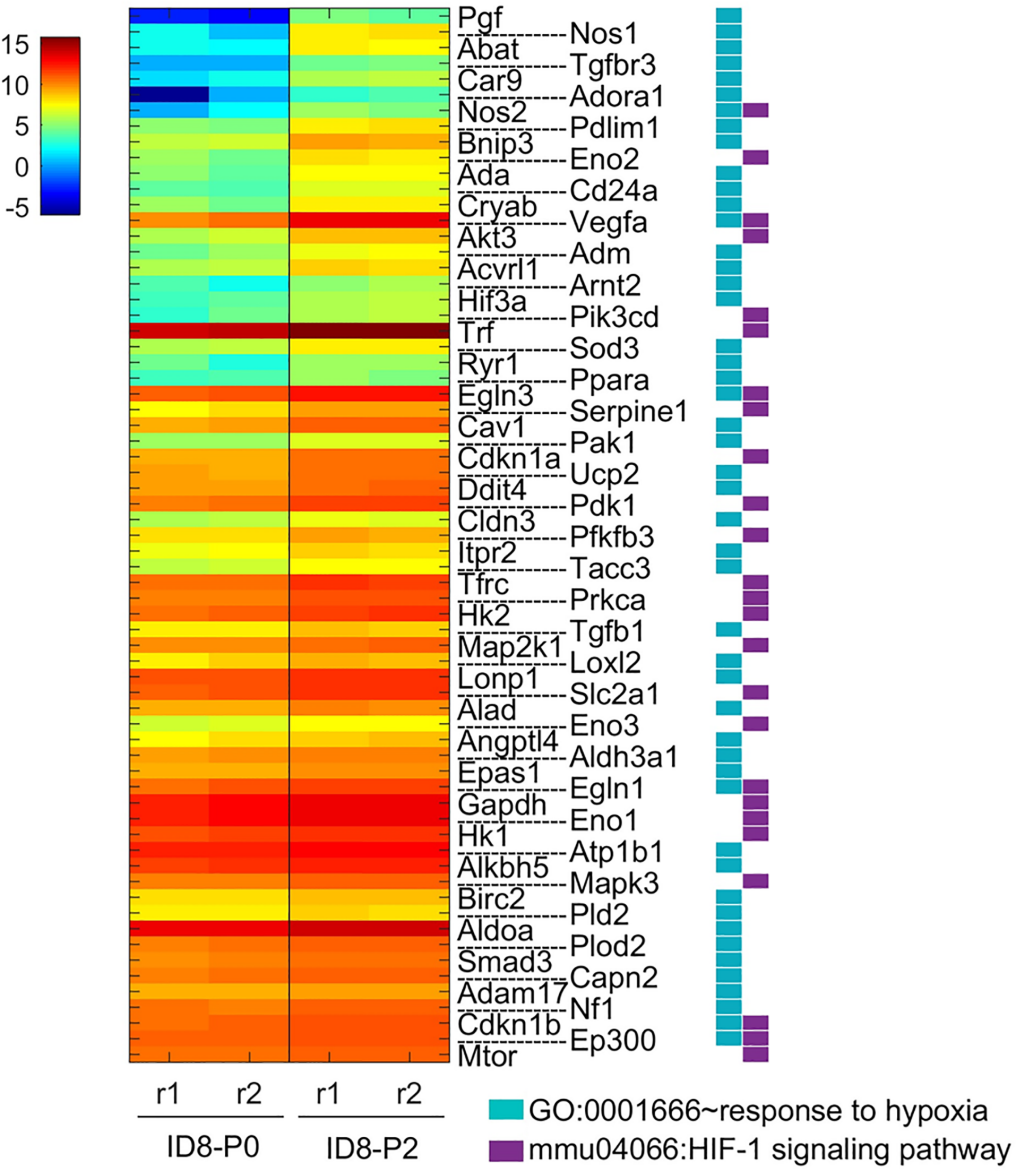
DEGs up-regulated in ID8-PW2 associated with hypoxia pathway. The gene expression heatmap (four samples) of 67 DEGs associated with GO:0001666~response to hypoxia and/or mmu04066:HIF-1 signaling pathway.

GO:0001666~response to hypoxia pathway identified 48 genes significantly up-regulated in ID8-P1 vs. ID8-P0 cells. The mmu04066:HIF-1 signaling pathway identified 25 genes regulated by HIF1. These genes are partially overlapping with 67 independent genes identified. We confirmed the differential expression of several HIF1α target genes Vegfa, Akt3, Trf, Eno2, Egln3, Nos2, Pdk1, Pkm, Ucp2, and Glut1 by Q-PCR and/or other biological assays **(Fig 8 and Table 1**).

**Table 1:** Confirmed hypoxic responsive genes up-regulated in ID8-P1 or–PW2 cells.

Gene name	RNAseq FC^1^	*P* value	Q-PCR Normalized^2^	Other assays
Vegfa	7.5	1.38 x 10^-68^	3.85±1.2	ELISA
Trf	3.5	1.56 x 10^-60^	8.0±10	
Akt3	6.7	3.61 x 10^-47^	2.35±0.2	pAkt Western [13]
Egln3	3.2	8.96 x 10^-44^	2.50±1.3	
Ucp2	2.4	9.62x10^-30^		Q-PCR (**Fig 6**)
Eno2	8.3	3.65 x 10^-28^	3.25±1.3	
Pdk1	2.2	2.64 x 10^-21^		Western (**Fig 4**)
Glut1 (Slc2A1)	1.7	3.01 x 10^-7^		Western [13]
Pkm	1.3	0.011838		Western (**Fig 4**)
Hif1a	0.84	0.016 (4115)		Western (no change; not shown)

^1^FC: fold change

^2^Fold change (ID8-P1 vs. P0); normalized to actin expression

We found that ID8-P1 cells produced 3.7-fold more VEGF than P0 cells when cells were attached (22.7 ng/ml ± 0.1 vs. 6.3 ± 0.2 ng/ml; all results were adjusted to the same cell numbers). More interestingly, when cell were cultured in suspension, they produced more VEGF (141± 5.3 and 36.6 ± 2.4 ng/ml from P1 and P0 cells, respectively), suggesting that compared to attached tumor cells, detached tumor cells may have enhanced tumor-promoting activities in at least certain aspects [13, 15].

### A subset development/differentiation, and/or stem cell related genes were upregulated in ID8-P1 or -P2 cells

**Fig 9:**
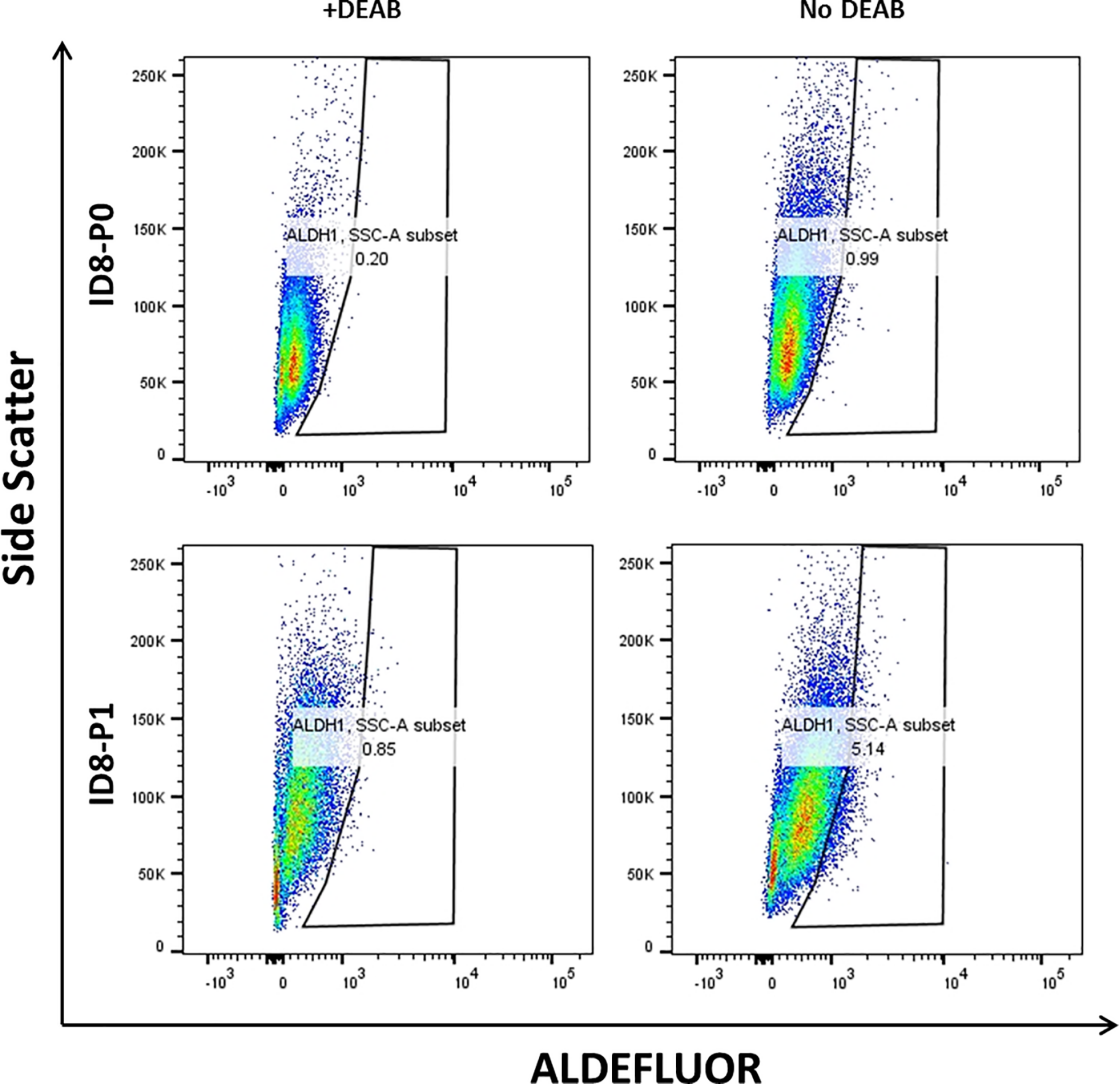
ID8-P1 cells had higher ALDH activity. Aldefluor assays were performed as detailed in Methods. Both ID8-P0 (upper row) and-P1 (lower row) were stained with ALDEFLUOR kit from StemCell Technologies. ALDH negative (left column) in the presence of the inhibitor DEAB; ALDH+ positive (right column) in the absence of the inhibitor DEAB.

As shown above, the genes/proteins detected to be up-regulated in ID8-P1 cells exhibited CSC-like activities. **Table 2**lists up-regulated genes involved in development and/or differentiation in ID8-P2/P1 cells vs. -P0 cells. Many of these genes have their roles implicated in CSC-like properties. In particular, the RNA-seq data showed that at least eight ALDH isoforms were up-regulated (**S2 Table**). We determined the total ALDH activity using the AldeFluor assays (**Fig 9**). ID8-P1 cells had significant higher ALDH activity than ID8-P0 cells.

**Table 2:** Genes related to CSC markers.

Gene	Fold	*P*	Test
**Zip4**	183	3.20x10^-217^	Expression and function [28]
**Gas6**	18.3	6.43x10^-165^	Q-PCR (**Fig 6**)
**Piwil2**	157	1.55x10^-117^	Western
**Gas7**	8.3	2.48x10^-100^	Q-PCR
**Ncam1**	6.2	2.76x10^-73^	
**Vegfa**	7.5	1.38x10^-68^	ELSA
**Kit (Cd117)**	43	3.40x10^-53^	FACS
**Akt3**	6.7	3.61x10^-47^	Western [13]
**Notch3**	11.2	3.03x10^-33^	Western
**Sox9**	2.6	1.04x10^-26^	Western and function [30]
**Cd24a**	7.7	2.10x10^-19^	
**Ctnnb1 (β- catenin)**	1.6	5.02x10^-14^	
**Epcam**	4.9	1.13x10^-8^	
**Abcc1**	1.5	2.91x10^-8^	
**CD44**	1.4	8.31x10^-6^	Western and IF
**Lgr5**	2.8	0.00091	

### Lipid metabolism was down-regulated in ID8-P1 and -P2 cells

Surprisingly, the major down-regulation pathways identified in ID8-PW2 vs. -P0 cells by RNA-seq were those involved in lipid metabolism, including cholesterol and sterol biosynthesis (**Fig 3D** and **S2 Table**). Several key enzymes involved in cholesterol and fatty acid synthesis, FDPS, Hmgcr and fatty acid synthase (Fasn) were both down- regulated in ID8-PW2 vs -P0 cells (0.24 x, *P* = 6.03 x 10^−27^; 0.52 x, *P* = 3.44 x 10^-15^; and 0.41 x, 6.60 x 10^-38^, *P* = 6.6 x 10^−38^, respectively). On the other hand, acetyl-CoA carboxylase β (Accb, 3.80x, *P* = 1.81x10^-15^), an enzyme which catalyzes the carboxylation of acetyl-CoA to malonyl-CoA, the rate-limiting step in fatty acid synthesis and malonyl-CoA-acyl carrier protein transacylase (Mcat, 2.0 x, *P* = 1.49x10^-5^) were up-regulated. It is also interesting to note that Lrp5, a gene which encodes a transmembrane low-density lipoprotein receptor that binds and internalizes ligands in the process of receptor-mediated endocytosis, was 6-fold upregulated in ID8-PW2 cells (*P* = 1.18 x10^-116^). This protein also acts as a co-receptor with Frizzled protein family members for transducing signals by Wnt proteins (39). In fact, several Wnt pathway genes were upregulated (see **S2 Table)** mmu04390:Hippo signaling pathway, Wnt5b, Wnt10b Fzd6, Fzd1,Wnt5a, and Wnt4). In addition, the very low-density lipoprotein receptor (Vldlr) was up-regulated (13.3 x, *P* = 4.94x10^-103^).

## Discussion

An ID8 syngeneic mouse EOC line was obtained through spontaneous transformation of normal ovarian surface epithelial cells from C57BL6 mice by repetitive passage *in vitro* [12]. Using mouse EOC cells has several advantages which would be difficult to achieve using human cells. 1) Human EOC cells from spontaneous transformation without transfection of immortalized and oncogenes are not presently available. Spontaneous transformation may better represent the highly heterogeneous human EOC than a genetic altered model. 2) The ID8 cells were obtained by *in vitro* passaging and are native to the tumor microenvironment. These cells may be more sensitive to the microenvironmental regulations. On the other hand, human cancer cell lines are isolated from human tumors and may be less sensitive to additional *in vivo* passages. This notion is supported by our finding that while ID8-P1 dramatically increased tumor aggressiveness (assessed by tumor/ascites formation time and peritoneal cavity floating tumor cells survival at early stage post i.p injection), ID8-P2 cells obtained by additional *in vivo* passage increased neither the aggressiveness *in vivo*, nor major survival signaling genes/pathways. 3) Mouse cells, as opposed to human cells, can be used in immune-competent tumor microenvironment, which is known to be critical for tumorigenesis [36, 37]. However, even though ID8 cells produce tumors resembling human high grade serous ovarian cancer (HGSOC) histologically [38], ID8 cells have similar oncogenic signaling pathways as those of human EOC cells [13, 39, 40], it still has limitations. Walton *et al*. have conducted whole exome sequencing of ID8 and found that there are no functional mutations in genes characteristic of human HGSOC (Trp53, Brca1, Brca2, Nf1, and Rb1), and p53 remained transcriptionally active. ID8 cells also lack other mutations typical of clear cell carcinoma (Arid1a, Pik3ca), low-grade serous carcinoma (Braf), endometrioid (Ctnnb1), or mucinous (Kras) carcinomas [41]. Genetically manipulated ID8 cells with loss of p53, with or without combination with other genes (pTen or Nf1) significantly reduced the median survival times of mice i.p. injected with these cells from ~ 90 days to 40–60 days [41, 42], which are very similar to ID8-P1 cells as we reported previously [13] and in the current study. As Walton’s ID8 genetic cell lines are superb for studying the roles of the particular genetic alterations detected in human HGSOC, it is likely the alterations acquired via *in vivo* passaging of ID8 cells through their interactions with the tumor microenvironment, as we reported here, are also related to the pathways regulated by these genes, reflecting the high heterogeneous, alternative, and flexible regulating mechanisms in cancer cells. Related to pTen, the PI3K-Akt pathway was upregulated in ID8-P1 vs. -P0 cells with Pik3cd and Akt3 genes upregulated (**S2 Table**). In contrast, Pik3ip1, a negative regulator the PI3K pathway [43], was down-regulated in ID8-P1 vs.–P0 cells (0.5.x, *P* = 2.6 x 10^-17^). Pten was also down regulated (0.84 x, *P* = 0.007). Regarding Nf1, a negative regulator of the Ras/MAPK signaling [44], several Ras-association domain family members, including Rassf1, 3, and 7 [45], were up-regulated (**S2 Table**). In addition, several Mapks were upregulated (Mapk3, 4, and 13, **S2 Table)**. Nevertheless, all model systems have their limitations and each of them may be more suitable to address specific issues. In fact, many results from studies of mouse-originated tumors have been successfully translated to human discoveries and clinical applications [46] and we have examined many genes revealed by the ID8 RNA-seq analyses in human EOC cells, supporting the value and relevance to human EOC studies.

The tumor microenvironment is comprised of the stromal cells, the extracellular matrix, soluble factors, and exosomes [47]. In addition, nutritional, regulatory, and energy factors, such as ATP, pH, and gas factors (e.g. O_2_, and NO) also play important roles. In addition to hypoxia, we found all three Nos genes (Nos1 to Nos3) were up-regulated (**S2 Table;** Nos3, 5.3x P = 0.01). Since the original establishment of ID8 cells [12], they have been passaged many times *in vivo*. However, the tumorigenic ability of ID8 cells have not been significantly changed (more than 100 mice have been tested in our labs), with consistent tumor/ascites formation time ~ 90 days as others reported [12, 38]. On the contrary, only one time *in vivo* passage has dramatically changed the gene expression landscape of the cells, as we showed here, supporting the roles of the tumor microenvironment. The cells and/or factors involved in the regulation warrant further investigation.

One of the unexpected findings is that the major down-regulated pathways are related to lipid metabolism, mainly to cholesterol and steroid hormone metabolisms. Lipids, including cholesterol and fatty acids, have been shown to play tumor-promoting roles in many cancers, including EOC [48–50]. Statins have been used in the management of several cancers [51, 52] Statins target HMG-CoA reductase (Hmgcr), which is the rate-limiting enzyme for cholesterol synthesis. However, data exist showing that statins may have negative impacts in cancer treatment. For example, statins actually promote invasive breast disease after long-term use [51]. Interestingly, Braicu *et al* have reported recently that HGSOC patients exhibit profound alterations in lipid metabolism. In particular, they have shown that ovarian cancer patients exhibited an overall reduction of most lipid classes in their serum as compared to a control group [53]. In addition, despite the overall reduction, there were also specific lipids showing elevation, and especially alterations in ceramide and triacylglycerol lipid species were dependent on their fatty acyl side chain composition. We have conducted lipidomic analyses for phospholipids, lysophospholipids, and triacylglycerols (TAGs) in less (ID8-P0) and highly aggressive (ID0-P1) EOC cell lines [54]. Our results show that the total lipid content analyses are not significantly different in ID8-P0 and -P1 cells [54]. However, when cells were cultured under detached condition, the total lipid mass was dramatically increased (2–3 fold, normalized to cell numbers) in both ID8-P0 and -P1 cells. In particular, TAG are dramatically increased (3.2–8.6 fold) when cells were cultured in detached conditions, which were the major contributors to the overall increases of the total lipid contents [54]. The findings reported by Braicu *et al* [53] are highly consistent with our previous [54] and current studies, suggesting the ID8 cell model reflects certain pathologic characteristics of human EOC.

In this work, we showed that more prominent changes in gene expression and/or cellular functions were observed in cells cultured in 3D or detached vs. 2D or attached conditions. In addition lipids, and TAGs in particular, are greatly increased when cells are detached [54]. For EOC, these observations are particularly relevant. Floating tumor cells are likely to be present at the very early stage of dissemination, as well as the very late stage when tumors and metastases are fully developed. Floating tumor cells tend to aggregate and are likely to be more drug-resistant; have higher motility and can develop new metastases; may interact with the stromal environment with different features from those solid tumor cells; and/or be primed for producing tumor- promoting factors more efficiently, as we showed for VEGF. Hence, targeting floating tumor cells, in addition to attached solid tumor cells, may become an important dimension of EOC therapeutics.

We have conducted more vigorous functional and signaling studies for ZIP4 and SOX9 [28, 30]. For other genes/proteins, more correlative data were collected and presented in the current work, which warrant further investigation. We have tested and confirmed up-regulation of ZIP4 expression in human EOC tissues [28]. Many of the identified genes/proteins in the RNA-seq data have been previously shown to be up-regulated in human EOC tissues, with potential clinical correlations. These include, but are not limited to, ALDH1, CD44, Epcam, VEGF, Notch3, Wnt/ β-catenin, GLUT1, and PDK1 [55–66]. Some of the other altered gene expressions are consistent with databases, such as Oncomine, which need to be further verified. This work is aiming for providing a landscape of how mouse EOC cells progress *in vivo*, which are likely to be extended to human EOC as we have shown in several our previous publications [13, 28, 30] and in the current work.

## Materials and methods

### Reagents, cell lines and culture

Oleoyl-LPA was from Avanti Polar Lipids (Birmingham, AL). Shikonin (SHK) and menadione (MD) were from Sigma (St. Louis, MO). Antibodies against p-FAK (Tyr397) and p-PXN (Tyr118) were from Cell Signaling (Boston, MA, USA). Antibodies against PKM2 and PDH1 were from Abcam. Anti-SOX9 antibody (Cat. Log # AB5535) was from EMD Millipore (Billerica, MA). The pair of PE01/PE04 cell lines were from Dr. Daniela Matei (Northwestern University); The ID8 and T29 cell lines were kind gifts from Dr. Paul F Terranova (University of Kansas Medical Center) and Dr. Jinsong Liu (M.D Anderson), respectively.

The ID8-P0, an ID8-luc cell line expressing both GFP and luciferase was established as described [39]. Different ID8-P1 and -P2 cell lines were established as previously described [13]. SKOV3 and SKOV3ip1 were from Dr. Mien-Chie Hung (University of Texas M.D. Anderson Cancer Center). HEY and HEY-1B cells were form Dr. Gorden Mills (MD Anderson). All cell lines were maintained in a humidified atmosphere at 37°C with 5% CO2. ID8 cells (mouse epithelial ovarian cancer cells) were maintained in high glucose DMEM containing 5% FBS (ATCC, Manassas, VA) and 100 μg/mL Penicillin/Streptomycin/ Amphotericin B (PSA). SKOV3 cells were cultured in Dulbecco's modified Eagle's medium/F12 supplemented with 4% FBS. HEY and HEY-1B cells were cultured in RPMI1640 with 5–10% FBS. PE01/PE04 cells were cultured in RPMI 1640 with glutamine, 10% FBS (ATCC, Manassas, VA), and 100 μg/mL Penicillin/Streptomycin/Amphotericin B (PSA). For serum starvation, cells were incubated in the basal medium without FBS or antibiotics. LPA treatment was performed in cells starved from serum for 16–24 hr.

### RNA-seq

ID8-P0 and–PW2 cells were cultured in suspension. After 24 hrs, cells were collected for RNA-seq analyses. A new and improved method for stranded whole transcriptome RNA-seq method was developed and described in detail previously [25]. Briefly, biological duplicates of cells (10^7^) were lysed and RNA was extracted according to manufacturer’s protocol (Qiagen RNeasy Mini kit). Total RNA was fractionated by size using ethanol concentration manipulations. The large RNA fraction (>200 nt) was fragmented prior to library construction. Ribosomal RNA was reduced by duplex specific nuclease (DSN) following limited hybridizations of both fractions and then amplified to add barcodes for multiplexing on the Illumina HiSeq2000 platform.

### Bioinformatics analyses

Demultiplexing RNA sequences were performed by CASAVA v1.8.2 and trimming was accomplished with Trimmomatic v0.22 with additional trimming by fastx_clipper v0.0.13.2. Read mapping was performed by tophat2 v2.0.6 to the mouse reference genome from ENSEMBL and bacterial genome with parameters, –b2-very-sensitive—read-edit-dist 2—max- multihits 100—library-type fr-secondstrand. Mapped reads were then summarized as gene expression for associated genes using custom perl scripts allowing no more than two mismatches. Differential analysis was performed with a published software edgeR [26] to evaluate the statistical significance of the difference and fold change (FC) between ID8-PW2 and -P0 for each individual gene. The q-values of genes were achieved by FDR adjustment on their p-values for multiple-test correction. If one gene has q-value less than 0.01 and linear fold change (FC) larger than 1.2 or less than 0.83, the gene was determined as differentially expressed gene (DEG). An online tool DAVID (https://david.ncifcrf.gov, v6.8) was used to perform enrichment analysis on DEGs for biological functions and KEGG pathways. A number of gene ontology (GO) functions, KEGG pathways, and other functional annotations were recognized as significantly over-represented (FDR-based multiple-test corrected p-values < 0.05) in either up- or down-regulated DEGs when comparing ID8-PW2 to ID8-P0.

### Stable cell lines

PE04-ZIP4-KO or PE01-ZIP4-OE cell lines were established as described [28]. The Dsp- KD cell lines were using The Dsp-KD cell lines were established by lentivirus infection of dsp- shRNA virus. Dsp-shRNA plasmid was from Origene (Cat# TL519230).

### Western blot analysis and quantitative-real time PCR (Q-PCR)

Western blot analyses were conducted using standard procedures and proteins were detected using primary antibodies and fluorescent secondary antibodies (IRDye 800CW- conjugated or IRDye 680-conjugated anti-species IgG, Li-Cor Biosciences, Lincoln, NE) as we described previously [13]. The fluorescent signals were captured on an Odyssey Infrared Imaging System (Li-Cor Biosciences, Lincoln, NE) with both 700- and 800-nm channels. Boxes were manually placed around each band of interest, and the software returned near- infrared fluorescent values of raw intensity with background subtraction (Odyssey 3.0 analytical software, Li-Cor Biosciences, Lincoln, NE). The protein MW marker used was the Pre-stained SDS-PAGE Standards, broad range (BIO_RAD, Cat. Log # 161–0318).

For quantitative real-time PCR (Q-PCR), mouse ID8 P0/P1 cells were seeded into 6-well plates in attached or low-attached plates in suspension, RNAs were extracted with the RNeasy mini kit (Qiagen, Valencia, CA) and reverse transcribed by M-MLV reverse transcriptase. Q- PCR was performed in a Light Cycler 480 (Roche, Indianapolis, IN) with a SYBR Green I Master Mix (Roche, Indianapolis, IN). mRNA abundance was normalized to GAPDH.

### ALDH activity assay

The Aldefluor assay (Stem Cell Technologies, Canada) was used to identify cell populations with ALDH enzymatic activity as described by MacDonagh *et al*. [67]. The assay was carried out according to manufacturer’s instructions. Briefly, cells (5 × 10^5^) were suspended in Aldefluor assay buffer containing activated Aldefluor reagent, BODIPY- aminoacetaldehyde (BAAA) for 45 min. The Aldefluor reagent is a fluorescent non-toxic ALDH substrate that freely diffuses into intact viable cells. In the presence of ALDH, BAAA is converted to BOPIDY-aminoacetate (BAA), which is retained within the cells expressing ALDH. A specific ALDH inhibitor, DEAB, was used to inhibit the BAAA-BAA conversion and acts as an internal negative control for background fluorescence. The brightly fluorescent ALDH1+ve cells were detected using the green fluorescence channel (520-540nm).

### Cell proliferation, anoikis-resistance, colony- and spheroid-formation assays

Cell proliferation was analyzed based on MTT hydrolysis using Cell Counting Kit-8 (Dojindo Molecular Technologies, Rockville, MA). Anoikis-resistance and soft agar colony assays were described in detail previously [13]. For spheroid formation, cells were re- suspended at 1×10^3^ to 1×10^4^ cells/mL in serum-free DMEM/F12 supplemented with 5 μg/mL insulin (Sigma), 20 ng/mL human recombinant epidermal growth factor (EGF; Invitrogen), 10 ng/mL basic fibroblast growth factor (bFGF; Invitrogen), and 0.4% bovine serum albumin (BSA; Sigma), followed by culturing in 24-or 96-well Ultra Low Attachment plates (Corning, NY). Spheroids were photographed after seven days in culture.

### Statistical analyses

The Student’s t-test was utilized to assess the statistical significance of the difference between two treatments. The asterisk rating system as well as quoting the *P* value in this study was * *P*< 0.05; ** *P*< 0.01; and *** *P*< 0.001. A *P* value of less than 0.05 was considered significant.

## Supporting information

S1 Fig Reproducibility of RNA-seq data. **A.** The scatter plot of gene expression between biological replicates, e.g. two replicates at P0. **B.** The scatter plot of gene expression between different conditions, e.g. one sample at P0 and the other one at PM2. **C.** Matrix of Pearson correlation coefficients between any two samples showing higher correlations between biological replicates than those cross different conditions.
(TIF)

S1 Table DEGs up-regulated in ID8-PW2 vs. -P0 associated with zinc ion binding. (DOCX)

S2 Table Gene ontologies (GOs), KEGG pathways and other functions significantly enriched in DEGs. The associated genes in the pathways with corresponding q-values are shown. The fold changes (ID8-P2 vs. ID8-P0) are enclosed in the bracket.
(DOCX)

S3 Table DEGs up-regulated in ID8-PW2 associated with pH pathway. The associated genes in the pathways with corresponding q-values are shown. The fold changes (ID8-P2 vs. ID8-P0) are enclosed in the bracket.
(DOCX)
