# Supplementary figures and images for: Changes in mRNA/protein expression and signaling pathways in in vivo passaged mouse ovarian cancer cells
Source: PLoS One. 2018 Jun 21;13(6):e0197404. doi: 10.1371/journal.pone.0197404 (PMC6013233; doi:10.1371/journal.pone.0197404)

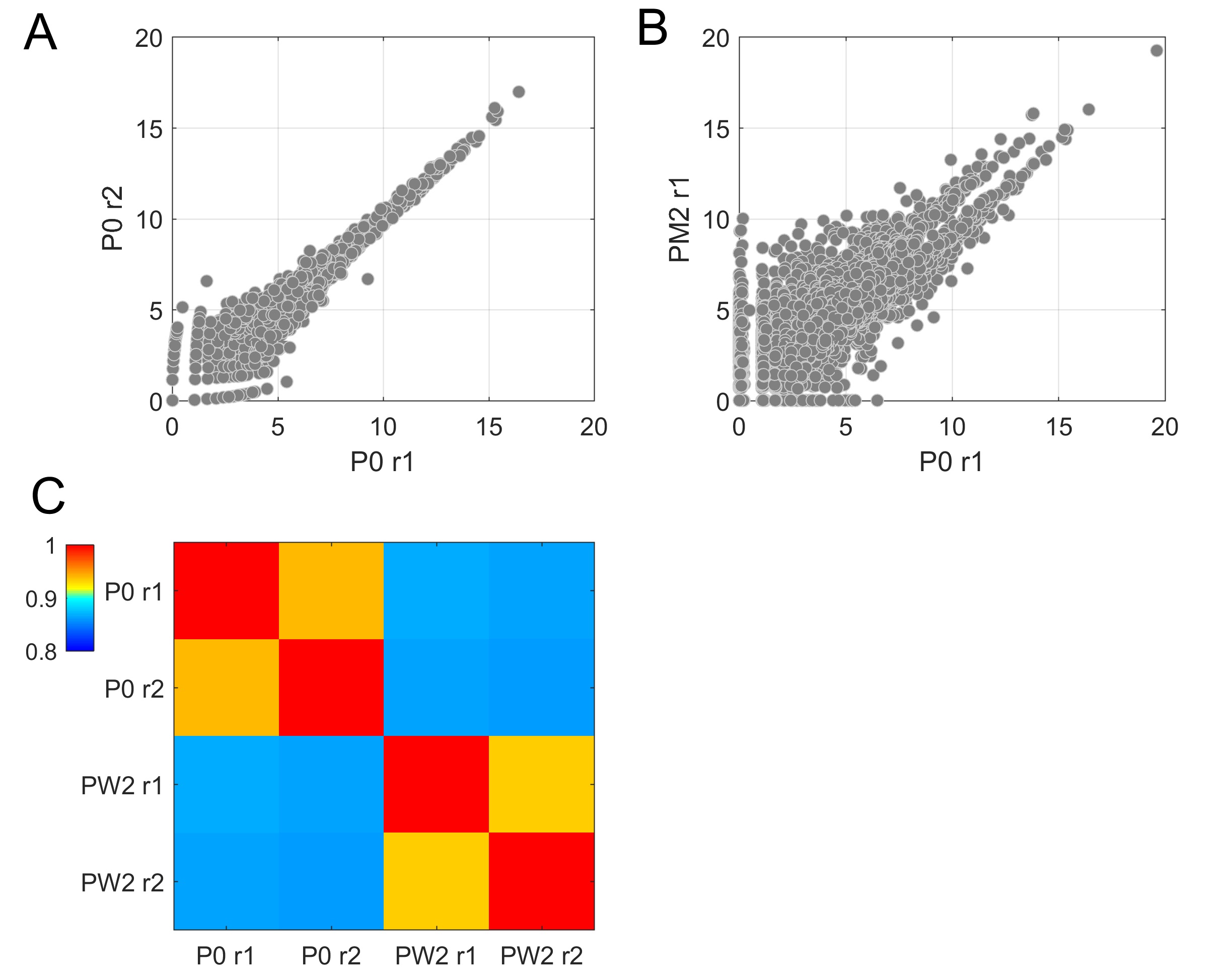

Supplement: S1 Fig — A. The scatter plot of gene expression between biological replicates, e.g. two replicates at P0. B. The scatter plot of gene expression between different conditions, e.g. one sample at P0 and the other one at PM2. C. Matrix of Pearson correlation coefficients between any two samples showing higher correlations between biological replicates than those cross different conditions. (TIF) [file pone.0197404.s001.tif]
