# Supplementary material for: Changes in mRNA/protein expression and signaling pathways in in vivo passaged mouse ovarian cancer cells
Source: PLoS One. 2018 Jun 21;13(6):e0197404. doi: 10.1371/journal.pone.0197404 (PMC6013233; doi:10.1371/journal.pone.0197404)
Supplement: S1 Table — (DOCX) [file pone.0197404.s002.docx]

**S1 Table. DEGs up-regulated in ID8-PW2 vs. -P0 associated with zinc ion binding**

| GOTERM_MF_DIRECT | q-value | Gene symbols with FC |
| --- | --- | --- |
| GO:0008270~zinc ion binding | 2.09E-02 | Pdlim1 (+8.7), Rad18 (+2.3), Rnf208 (+85.3), Mical2 (+1.7), Rnf181 (+1.5), Lonrf1 (+1.9), Mmp17 (+8.1), Nr1d1 (+1.9), 2010111I01Rik (+1.4), Bard1 (+2.7), Sod3 (+3.4), Cpn1 (+5.3), Adam33 (+10.6), Zfand2a (+1.4), Enpp1 (+2.2), Car6 (+14.4), Zcrb1 (+1.4), Yaf2 (+1.9), G2e3 (+2.0), Aire (+29.4), Agbl5 (+2.1), Dtx1 (+24.5), Cblb (+1.3), Pdlim4 (+15.7), Esr1 (+1.7), Pcgf3 (+1.5), Crip3 (+83.2), Zfr2 (+3.8), Trim14 (+3.4), L3mbtl2 (+1.4), Uhrf2 (+1.3), Ace (+7.4), Sqstm1 (+1.2), Zcchc11 (+1.3), Rnf43 (+6.7), Ttc3 (+1.6), Car12 (+2.1), Mt2 (+25.4), Ing4 (+1.4), Rsf1 (+1.3), Tk1 (+2.6), Apobec3 (+2.0), Car9 (+17.1), Pcgf5 (+1.7), Ppara (+3.3), Cul9 (+8.3), Rnf19a (+2.1), Nr5a1 (+1.4), Trim30a (+54.8), Brd1 (+1.4), Gata4 (+1.6), Vdr (+2.0), Mt1 (+3.0), Dzip3 (+1.9), Adamts4 (+6.6), Usp22 (+1.4), Cxxc4 (+2.5), Map3k1 (+1.6), Arih1 (+1.3), Parp1 (+1.6), Birc5 (+2.3), Birc2 (+1.5), March6 (+1.6), Enpp2 (+4.9), Ubr5 (+1.3), Cda (+7.7), Pja1 (+1.3), Fus (+1.7), Nrap (+55.7), Rnf122 (+1.9), Chd3 (+1.5), Uhrf1 (+2.0), Cryz (+2.0), Kdm5b (+1.3), Nr1i2 (+7.6), Mme (+4.6), Brca1 (+1.8), Hivep1 (+1.3), Zcchc14 (+1.3), Rara (+1.5), Kcmf1 (+1.3), Smad3 (+1.4), Mmel1 (+9.1), Rnf141 (+2.3), Mta3 (+1.7), Dtnb (+1.6), Zfand3 (+1.3), Rnf182 (+12.1), Bnc2 (+1.5), Zmiz1 (+1.5), Mdm4 (+1.6), Crip1 (+1.5), Lonrf3 (+2.2), Traf1 (+23.2), Micall1 (+1.5), Adamts10 (+4.1), Lims2 (+59.6), Trip12 (+1.2), Mdm2 (+1.4), Phf19 (+5.3), Rnf2 (+1.5), Pdlim2 (+2.2), Scaf11 (+1.5), Zfr (+1.4), Anpep (+11.7), Enpep (+23.6), Pappa2 (+9.2), Mmp19 (+2.5), Tet1 (+5.7), Ski (+1.3), Dtx3 (+1.9), Tet2 (+2.0), Nsd1 (+1.4), Lhx1 (+80.0), Rarg (+1.6), Ep300 (+1.3), Neil2 (+6.7), Qpct (+153.1), Sp140 (+4.8), Ablim1 (+2.2), Pxn (+1.3), Rnf145 (+1.6), Thra (+1.6), Prkca (+1.9), Micall2 (+2.4), Rora (+2.0), Kdm4b (+1.4), Ada (+7.8), Pdlim7 (+1.5), Nsmce2 (+1.4), Nfx1 (+1.3), Zcwpw1 (+3.1), Gata5 (+6.0), Trim23 (+1.4), Kdm5a (+1.4), Alad (+1.7) |
