# Supplementary material for: Changes in mRNA/protein expression and signaling pathways in in vivo passaged mouse ovarian cancer cells
Source: PLoS One. 2018 Jun 21;13(6):e0197404. doi: 10.1371/journal.pone.0197404 (PMC6013233; doi:10.1371/journal.pone.0197404)
Supplement: S2 Table — The associated genes in the pathways with corresponding q-values are shown. The fold changes (ID8-P2 vs. ID8-P0) are enclosed in the bracket. (DOCX) [file pone.0197404.s003.docx]

**S2 Table. Gene Ontologies (GOs), KEGG pathways and other functions enriched in DEGs**

| **Proliferation/Apoptosis** | **q-value** | **Gene symbols with fold change (FC)** |
| --- | --- | --- |
| GO:0007049~cell cycle | 1.14E-07 | Sept9 (+1.3), Anapc15 (+2.0), Fbxo5 (+2.7), Cit (+2.4),  Pdcd6ip (+1.3), Kif23 (+2.6), 4632434I11Rik (+2.1), Ddit3  (+1.5), Cks2 (+2.5), Gmnn (+2.2), Cdc20 (+2.1), Ndc80  (+2.0), Dclre1a (+1.5), Mis18bp1 (+2.4), Maea (+1.4),  Fam64a (+3.6), Uhrf2 (+1.3), Mapk4 (+2.6), Nusap1 (+2.0),  Cep55 (+1.9), Ska2 (+1.6), Ing4 (+1.4), Dlgap5 (+3.3), Prc1  (+2.1), Triobp (+1.4), Aspm (+1.6), Kntc1 (+2.0), Rad21  (+1.6), Brsk1 (+27.7), Knstrn (+2.0), Prkcd (+1.5), Syce2  (+3.4), Cdk5 (+1.3), Mapk3 (+1.5), Plk1 (+3.1), Dscc1  (+5.1), Steap3 (+7.9), Ncapd2 (+1.6), Usp22 (+1.4), Fanci  (+2.5), Aurkb (+2.7), Birc5 (+2.3), Cenpe (+1.8), Sgol1  (+2.8), Ppp1cb (+1.3), Ticrr (+3.3), Cdc45 (+2.4), Sik1  (+1.6), Sept10 (+1.3), Ccng2 (+1.6), Nek2 (+2.5), Hjurp  (+1.7), Ppp1cc (+1.4), Cdkn1b (+1.3), Uhrf1 (+2.0), Setd8  (+1.6), Anapc5 (+1.3), Mastl (+1.9), Nedd9 (+2.0), Dab2ip  (+1.5), Brca1 (+1.8), Ska3 (+2.6), Ccnb2 (+2.2), Mapk13  (+36.7), Cdkn1a (+2.6), Prr5 (+4.2), Aurka (+2.5), Dynlt3  (+1.3), Kif11 (+1.4), E2f7 (+3.3), Ccnd2 (+2.8), Ncaph  (+1.8), Ccnb1 (+1.9), Mki67 (+2.3), Bub1b (+2.3), Gsg2  (+6.7), Nipbl (+1.4), Tet2 (+2.0), Ep300 (+1.3), Arl2 (+1.4),  Cdk1 (+1.8), Rassf1 (+1.8), Gnai2 (+1.3), Camk1 (+1.6),  Tpx2 (+1.9), Fancd2 (+5.7), Gadd45a (+2.1), Clasp2 (+1.5),  Dixdc1 (+8.7), Marveld1 (+1.3), Kctd11 (+2.1), Ube2c (+2.0),  Spag5 (+1.9), Chek2 (+2.4), Snx18 (+1.5), Foxn3 (+1.4),  Txnip (+1.6), Nsmce2 (+1.4), Pmp22 (+4.0), Tacc1 (+1.3),  Racgap1 (+2.6), Arl8b (+1.3), Cul4b (+1.5), Anln (+2.0),  Ccna2 (+2.2), Pkmyt1 (+2.3), Rab11a (+1.3) |
| Cell division | 1.50E-05 | Sept9 (+1.3), Ppp1cc (+1.4), Anapc15 (+2.0), Fbxo5 (+2.7),  Setd8 (+1.6), Cit (+2.4), Anapc5 (+1.3), Pdcd6ip (+1.3),  Kif23 (+2.6), Nedd9 (+2.0), Mastl (+1.9), Ska3 (+2.6), Cks2  (+2.5), Ccnb2 (+2.2), Cdc20 (+2.1), Ndc80 (+2.0), Aurka  (+2.5), Dclre1a (+1.5), Mis18bp1 (+2.4), Kif11 (+1.4), Dynlt3  (+1.3), Maea (+1.4), Fam64a (+3.6), Ccnd2 (+2.8), Ncaph  (+1.8), Ccnb1 (+1.9), Nusap1 (+2.0), Bub1b (+2.3), Cep55  (+1.9), Ska2 (+1.6), Prc1 (+2.1), Triobp (+1.4), Cdk1 (+1.8),  Gnai2 (+1.3), Aspm (+1.6), Tpx2 (+1.9), Kntc1 (+2.0),  Clasp2 (+1.5), Rad21 (+1.6), Knstrn (+2.0), Syce2 (+3.4),  Ube2c (+2.0), Chek2 (+2.4), Tacc3 (+2.2), Kifc5b (+3.7),  Spag5 (+1.9), Snx18 (+1.5), Cdk5 (+1.3), Plk1 (+3.1),  Nsmce2 (+1.4), Ncapd2 (+1.6), Racgap1 (+2.6), Tacc1  (+1.3), Arl8b (+1.3), Aurkb (+2.7), Birc5 (+2.3), Cenpe  (+1.8), Sgol1 (+2.8), Ppp1cb (+1.3), Anln (+2.0), Cdc45  (+2.4), Sept10 (+1.3), Ccng2 (+1.6), Ccna2 (+2.2), Nek2  (+2.5) |
| Mitosis | 2.13E-05 | Anapc15 (+2.0), Fbxo5 (+2.7), Setd8 (+1.6), Cit (+2.4),  Anapc5 (+1.3), Kif23 (+2.6), Nedd9 (+2.0), Mastl (+1.9), |

|  |  | Ska3 (+2.6), Ccnb2 (+2.2), Cdc20 (+2.1), Ndc80 (+2.0),  Aurka (+2.5), Dclre1a (+1.5), Mis18bp1 (+2.4), Kif11 (+1.4),  Dynlt3 (+1.3), Fam64a (+3.6), Ncaph (+1.8), Ccnb1 (+1.9),  Nusap1 (+2.0), Bub1b (+2.3), Cep55 (+1.9), Ska2 (+1.6),  Triobp (+1.4), Cdk1 (+1.8), Aspm (+1.6), Tpx2 (+1.9), Kntc1  (+2.0), Clasp2 (+1.5), Rad21 (+1.6), Knstrn (+2.0), Ube2c  (+2.0), Chek2 (+2.4), Tacc3 (+2.2), Kifc5b (+3.7), Spag5  (+1.9), Snx18 (+1.5), Plk1 (+3.1), Nsmce2 (+1.4), Ncapd2  (+1.6), Arl8b (+1.3), Aurkb (+2.7), Birc5 (+2.3), Cenpe  (+1.8), Sgol1 (+2.8), Anln (+2.0), Ccng2 (+1.6), Ccna2  (+2.2), Nek2 (+2.5) |
| --- | --- | --- |
| GO:0051301~cell division | 8.25E-05 | Sept9 (+1.3), Ppp1cc (+1.4), Anapc15 (+2.0), Fbxo5 (+2.7),  Setd8 (+1.6), Cit (+2.4), Anapc5 (+1.3), Pdcd6ip (+1.3),  Kif23 (+2.6), Nedd9 (+2.0), Mastl (+1.9), Ska3 (+2.6), Cks2  (+2.5), Ccnb2 (+2.2), Cdc20 (+2.1), Ndc80 (+2.0), Aurka  (+2.5), Dclre1a (+1.5), Map4 (+1.3), Mis18bp1 (+2.4), Kif11  (+1.4), Dynlt3 (+1.3), Maea (+1.4), Fam64a (+3.6), Ccnd2  (+2.8), Ncaph (+1.8), Ccnb1 (+1.9), Nusap1 (+2.0), Bub1b  (+2.3), Cep55 (+1.9), Ska2 (+1.6), Prc1 (+2.1), Triobp  (+1.4), Cdk1 (+1.8), Gnai2 (+1.3), Aspm (+1.6), Tpx2 (+1.9),  Kntc1 (+2.0), Clasp2 (+1.5), Rad21 (+1.6), Knstrn (+2.0),  Syce2 (+3.4), Ube2c (+2.0), Spag5 (+1.9), Chek2 (+2.4),  Kifc5b (+3.7), Snx18 (+1.5), Cdk5 (+1.3), Plk1 (+3.1),  Nsmce2 (+1.4), Ncapd2 (+1.6), Racgap1 (+2.6), Tacc1  (+1.3), Arl8b (+1.3), Birc5 (+2.3), Aurkb (+2.7), Cenpe  (+1.8), Sgol1 (+2.8), Ppp1cb (+1.3), Anln (+2.0), Cdc45  (+2.4), Sept10 (+1.3), Ccng2 (+1.6), Ccna2 (+2.2), Nek2  (+2.5), Kif14 (+2.2) |
| Apoptosis | 2.12E-03 | Rassf7 (+2.4), Pdcd6ip (+1.3), Rtkn (+1.8), 4632434I11Rik  (+2.1), Dab2ip (+1.5), Pmaip1 (+2.1), Gadd45b (+1.6), Ddit3  (+1.5), Egln3 (+3.2), Plscr3 (+1.5), Mtch1 (+1.4), Foxo1  (+1.6), Gapdh (+1.5), Fam162a (+1.9), Casp7 (+2.1), Tial1  (+1.5), G2e3 (+2.0), Aplp1 (+1.5), Puf60 (+1.3), Sh3kbp1  (+2.2), Traf1 (+23.2), Vdac1 (+1.4), Dab2 (+1.6), Hip1  (+1.5), Dap (+2.5), Bag6 (+1.6), Cadm1 (+1.5), Pak1 (+2.6),  Ero1l (+3.6), Prune2 (+1.8), Hif3a (+4.4), Ngfrap1 (+2.1),  Lgals1 (+1.9), Bub1b (+2.3), Sqstm1 (+1.2), Nisch (+1.4),  Ing4 (+1.4), Prelid1 (+1.5), Cdk1 (+1.8), Shf (+2.5), Ddit4  (+2.3), Ifit2 (+4.8), Bok (+1.5), Tpx2 (+1.9), Tmem173  (+1.7), Bnip3 (+8.4), Cflar (+1.4), Rad21 (+1.6), Prkca  (+1.9), Vil1 (+9.7), Prkcd (+1.5), Fgfr3 (+3.6), Dedd (+1.6),  Chek2 (+2.4), Stk24 (+1.4), Cdk5 (+1.3), Mapk3 (+1.5),  Pycard (+4.3), Steap3 (+7.9), Ebag9 (+1.4), Arl6ip1 (+1.3),  Mef2a (+1.4), Fem1b (+1.3), Ppp1r13l (+1.7), Tmbim6  (+1.4), Birc5 (+2.3), Bcl2l2 (+1.3), Birc2 (+1.5), Kank2  (+1.7), Fastk (+1.7), Pea15a (+1.6) |
| mmu04390:Hippo signaling pathway | 5.97E-03 | Ppp1cc (+1.4), Dlg3 (+4.5), Csnk1e (+1.4), Tcf7 (+28.3),  Tgfb1 (+1.9), Dlg4 (+2.2), Wnt5b (+2.7), Wnt10b (+8.9),  Rassf1 (+1.8), Serpine1 (+3.1), Smad3 (+1.4), Smad1  (+6.1), Bmp7 (+3.3), Tcf7l2 (+1.7), Ywhaz (+1.6), Fzd6  (+1.9), Ctnnb1 (+1.6), Bmpr2 (+1.4), Dlg2 (+12.0), Fzd1  (+2.7), Wnt5a (+1.6), Frmd6 (+1.9), Birc5 (+2.3), Afp  (+12.3), Gsk3b (+1.4), Ppp1cb (+1.3), Ccnd2 (+2.8), Snai2  (+2.2), Fgf1 (+3.0), Wnt4 (+6.3) |
| mmu04110:Cell cycle | 6.70E-03 | Cdkn1b (+1.3), Tgfb1 (+1.9), Ep300 (+1.3), Cdk1 (+1.8), |

|  |  | Anapc5 (+1.3), Gadd45a (+2.1), Gadd45b (+1.6), Smad3  (+1.4), Rad21 (+1.6), Ccnb2 (+2.2), Cdc20 (+2.1), Cul1  (+1.3), Chek2 (+2.4), Cdkn1a (+2.6), Ywhaz (+1.6), Plk1  (+3.1), Ttk (+3.9), Espl1 (+2.5), Mdm2 (+1.4), Gsk3b (+1.4),  Ccnd2 (+2.8), Cdc45 (+2.4), Ccnb1 (+1.9), Ccna2 (+2.2),  Bub1b (+2.3), Pkmyt1 (+2.3) |
| --- | --- | --- |
| mmu05200:Pathways in cancer | 8.58E-03 | Cdkn1b (+1.3), Tcf7 (+28.3), Tgfb1 (+1.9), Fgf11 (+5.2),  Wnt10b (+8.9), Rara (+1.5), Plcb4 (+1.9), Adcy6 (+1.3),  Egln3 (+3.2), Smad3 (+1.4), Cks2 (+2.5), Slc2a1 (+1.7),  Ptger4 (+2.7), Fn1 (+1.7), Runx1 (+1.3), Foxo1 (+1.6),  Pdgfa (+2.6), Cdkn1a (+2.6), Pdgfra (+5.6), Ccdc6 (+1.4),  Plekhg5 (+1.4), Fzd6 (+1.9), Ctnnb1 (+1.6), Cblb (+1.3),  Traf1 (+23.2), Lamb1 (+1.4), Mdm2 (+1.4), Adcy7 (+3.9),  Pgf (+180.4), Kitl (+1.5), Fgf1 (+3.0), Wnt4 (+6.3), Gna12  (+1.6), Ep300 (+1.3), Gnai2 (+1.3), Wnt5b (+2.7), Rassf1  (+1.8), Kit (+43.4), Arnt2 (+4.5), Vegfa (+7.5), Prkca (+1.9),  Fgfr1 (+1.6), Fgfr3 (+3.6), Tcf7l2 (+1.7), Adcy3 (+10.4),  Mapk3 (+1.5), Mtor (+1.3), Gnb4 (+23.4), Msh3 (+1.6),  Egln1 (+1.6), Fzd1 (+2.7), Map2k1 (+1.8), Akt3 (+6.7),  Pik3cd (+3.9), Wnt5a (+1.6), Birc5 (+2.3), Birc2 (+1.5),  Gsk3b (+1.4), Epas1 (+1.6), Nos2 (+12.7), Csf1r (+28.9) |
| mmu04115:p53 signaling pathway | 1.12E-02 | Steap3 (+7.9), Sesn3 (+2.5), Mdm4 (+1.6), Cdk1 (+1.8),  Serpine1 (+3.1), Rrm2b (+1.4), Mdm2 (+1.4), Pmaip1 (+2.1),  Gadd45a (+2.1), Gtse1 (+2.0), Gadd45b (+1.6), Ccnd2  (+2.8), Ccnb2 (+2.2), Ccnb1 (+1.9), Ccng2 (+1.6), Chek2  (+2.4), Cdkn1a (+2.6) |
| mmu04068:FoxO signaling pathway | 2.17E-02 | Cdkn1b (+1.3), Csnk1e (+1.4), Tgfb1 (+1.9), Prkag1 (+1.5),  Ep300 (+1.3), Bcl6 (+1.4), Gadd45a (+2.1), Prkaa1 (+1.7),  Bnip3 (+8.4), Gadd45b (+1.6), Smad3 (+1.4), Mapk13  (+36.7), Ccnb2 (+2.2), Pck2 (+1.4), Foxo1 (+1.6), Cdkn1a  (+2.6), Mapk3 (+1.5), Plk1 (+3.1), Fbxo32 (+1.3), Pik3cd  (+3.9), Akt3 (+6.7), Map2k1 (+1.8), Mdm2 (+1.4), Ccnd2  (+2.8), Ccnb1 (+1.9), Ccng2 (+1.6) |
| GO:0006915~apoptotic process | 4.58E-02 | Rassf7 (+2.4), Aldoc (+1.5), Pdcd6ip (+1.3), Rtkn (+1.8),  4632434I11Rik (+2.1), Trpv2 (+52.4), Dab2ip (+1.5),  Pmaip1 (+2.1), Lsp1 (+278.9), Gadd45b (+1.6), Ddit3 (+1.5),  Egln3 (+3.2), Plscr3 (+1.5), Mtch1 (+1.4), Foxo1 (+1.6), Cul1  (+1.3), Gapdh (+1.5), Fam162a (+1.9), Casp7 (+2.1), Tial1  (+1.5), G2e3 (+2.0), Aplp1 (+1.5), Puf60 (+1.3), Traf1  (+23.2), Sh3kbp1 (+2.2), Vdac1 (+1.4), Dab2 (+1.6), Hip1  (+1.5), Dap (+2.5), Bag6 (+1.6), Cadm1 (+1.5), Pak1 (+2.6),  Ero1l (+3.6), Prune2 (+1.8), Hif3a (+4.4), Ngfrap1 (+2.1),  Lgals1 (+1.9), Bub1b (+2.3), Sqstm1 (+1.2), Nisch (+1.4),  Ing4 (+1.4), Prelid1 (+1.5), Ep300 (+1.3), Cdk1 (+1.8), Shf  (+2.5), Ifit2 (+4.8), Ddit4 (+2.3), Bok (+1.5), Tpx2 (+1.9),  Tmem173 (+1.7), Bnip3 (+8.4), Cflar (+1.4), Rad21 (+1.6),  Prkca (+1.9), Vil1 (+9.7), Prkcd (+1.5), Fgfr3 (+3.6), Dedd  (+1.6), Chek2 (+2.4), Stk24 (+1.4), Cdk5 (+1.3), Mapk3  (+1.5), Pycard (+4.3), Steap3 (+7.9), Ebag9 (+1.4), Arl6ip1  (+1.3), Mef2a (+1.4), Fem1b (+1.3), Ppp1r13l (+1.7), Birc5  (+2.3), Tmbim6 (+1.4), Bcl2l2 (+1.3), Birc2 (+1.5), Aatk  (+14.4), Kank2 (+1.7), Cul7 (+1.9), Fastk (+1.7), Pea15a  (+1.6) |

| **Cytoskeleton** | **q-value** | **Gene symbols with fold change (FC)** |
| --- | --- | --- |
| GO:0005856~cytoskeleton | 2.20E-08 | Sept9 (+1.3), Eml1 (+4.9), Pdlim1 (+8.7), Twf1 (+1.4), Rad18  (+2.3), Rassf3 (+2.5), Rassf7 (+2.4), Ptpn7 (+5.5), Fbxo5  (+2.7), Acot13 (+1.4), Pdcd6ip (+1.3), Kif23 (+2.6), Fam96b  (+1.9), Disc1 (+22.4), Tbce (+1.6), Map1a (+3.9), Enc1  (+2.2), Dtl (+2.4), Mapk1ip1 (+1.8), Parvb (+12.8), Ky (+3.2),  Cdc20 (+2.1), Shroom1 (+6.7), Ccdc6 (+1.4), Bsn (+3.6),  Myzap (+3.2), Sptb (+2.6), Lrmp (+6.1), Arl6ip5 (+1.2), Agbl5  (+2.1), Arap3 (+6.4), Mylk (+2.2), Tubb6 (+1.8), Maea (+1.4),  Trib2 (+1.6), Kitl (+1.5), Cttn (+1.4), Nusap1 (+2.0), Ndrg1  (+3.5), Nos1 (+63.9), Ctnnal1 (+1.6), Cep55 (+1.9), Kif21a  (+1.9), Des (+9.1), Cntln (+2.3), Ska2 (+1.6), Eml4 (+1.4),  Tacc2 (+1.3), Dlgap5 (+3.3), Prc1 (+2.1), Triobp (+1.4),  Myo7b (+15.4), Aspm (+1.6), Kntc1 (+2.0), Gtse1 (+2.0),  Myo18a (+1.3), Tuba8 (+83.7), Rnf19a (+2.1), Tpm2 (+45.8),  Pacsin2 (+1.6), Knstrn (+2.0), Brsk1 (+27.7), Map7 (+8.8),  Map1b (+1.3), Vil1 (+9.7), Ccdc38 (+52.3), Plekhg6 (+5.2),  Tppp (+5.0), Dnm1 (+3.6), Mapk3 (+1.5), Cdk5 (+1.3), Plk1  (+3.1), Map3k1 (+1.6), Bbs4 (+2.4), Map2k1 (+1.8), Rmdn2  (+2.1), Birc5 (+2.3), Avil (+9.6), Aurkb (+2.7), Cenpe (+1.8),  Sgol1 (+2.8), Ckap4 (+1.8), Cep57l1 (+2.6), Sept10 (+1.3),  Add1 (+1.3), Ank2 (+2.0), Cul7 (+1.9), Baiap2 (+1.3), Nek2  (+2.5), Coro2b (+6.2), Clip2 (+1.9), Afap1 (+2.3), Mybpc3  (+7.6), Nedd9 (+2.0), Tubg2 (+52.6), Mastl (+1.9), Ska3  (+2.6), Sncg (+62.7), Smtn (+1.4), Dync1i1 (+1.6), Klhl2  (+1.7), Cotl1 (+2.0), Cep57 (+1.5), Atf4 (+1.5), Dennd2a  (+7.9), Twf2 (+1.8), Aurka (+2.5), Ctnnb1 (+1.6), Map4  (+1.3), Kif22 (+1.9), Kif11 (+1.4), Dynlt3 (+1.3), Tmod3  (+1.4), Sh3kbp1 (+2.2), Frmd6 (+1.9), Sorbs3 (+1.6), Pdlim2  (+2.2), Add3 (+1.6), Hspa2 (+1.8), Baiap2l1 (+1.2), Ccnb1  (+1.9), Hspb1 (+2.1), D430042O09Rik (+1.5), Mtss1 (+1.9),  Gsg2 (+6.7), Dsp (+55.4), Ppp1r18 (+1.4), Cep170 (+1.4),  Frmd4a (+1.9), Dpysl2 (+1.3), Myo7a (+3.1), Cdk1 (+1.8),  Arl2 (+1.4), Rassf1 (+1.8), Gnai2 (+1.3), Sgcb (+2.2), Ablim1  (+2.2), Tpx2 (+1.9), Rai14 (+1.9), Clasp2 (+1.5), Pxn (+1.3),  Nckap5l (+2.8), Micall2 (+2.4), Marveld1 (+1.3), Tpm1 (+1.3),  Kif27 (+7.4), Spag5 (+1.9), Klhl3 (+30.3), Ckap2l (+2.1),  Pick1 (+1.4), Pdlim7 (+1.5), Vim (+1.4), Ivns1abp (+1.8),  Tacc1 (+1.3), Racgap1 (+2.6), Arl8b (+1.3), Cct5 (+1.3), Def6  (+121.2), Palld (+1.8), Dnah17 (+4.6), Anln (+2.0), Soga2  (+2.1), Camsap3 (+83.8), Cdc42ep2 (+6.0), Coro1b (+1.3),  Kif20a (+2.3), Kif4 (+1.8), Kif14 (+2.2) |
| GO:0042060~wound healing | 3.65E-05 | Mtor (+1.3), Dsp (+55.4), Nf1 (+1.3), B4galt1 (+1.3), Pecam1  (+16.3), Arhgef19 (+1.8), Map3k1 (+1.6), Tgfb1 (+1.9), Hpse  (+4.9), Wnt5a (+1.6), Sparc (+2.0), Fcgr4 (+16.2), Wnt5b  (+2.7), Cdh3 (+3.1), Dcbld2 (+1.9), Serpine1 (+3.1), Col3a1  (+8.2), Ppara (+3.3), Pak1 (+2.6), Elk3 (+1.6), Dcn (+1.2),  Fn1 (+1.7), Tpm1 (+1.3), Pdgfra (+5.6), Cav1 (+2.7), Coro1b  (+1.3) |
| GO:0003779~actin binding | 1.26E-04 | Nrap (+55.7), Twf1 (+1.4), Coro2b (+6.2), Afap1 (+2.3),  Mybpc3 (+7.6), Mical2 (+1.7), Myh14 (+15.9), Myh7b (+51.6),  Phactr1 (+7.5), Lsp1 (+278.9), Map1a (+3.9), Enc1 (+2.2),  Dbn1 (+1.4), Parvb (+12.8), Klhl2 (+1.7), Shroom1 (+6.7), |

|  |  | Myoz2 (+91.0), Cotl1 (+2.0), Twf2 (+1.8), Sptb (+2.6), Tns1  (+28.1), Coro2a (+16.1), Tmod3 (+1.4), Hip1 (+1.5), Myo5b  (+3.6), Mylk (+2.2), Myh10 (+20.6), Actn3 (+4.2), Maea  (+1.4), Add3 (+1.6), Myo1h (+2.7), Baiap2l1 (+1.2), Fmnl1  (+2.2), Mtss1 (+1.9), Pof1b (+4.0), Ppp1r18 (+1.4), Ncald  (+4.7), Fxyd5 (+1.8), Cald1 (+1.3), Myo7a (+3.1), Triobp  (+1.4), Tnni1 (+14.4), Myo7b (+15.4), Ablim1 (+2.2), Vill  (+2.0), Tnni3 (+8.9), Tpm2 (+45.8), Dixdc1 (+8.7), Map1b  (+1.3), Vil1 (+9.7), Tpm1 (+1.3), Klhl3 (+30.3), Pick1 (+1.4),  Gmfg (+56.1), Avil (+9.6), Palld (+1.8), Nos2 (+12.7), Anln  (+2.0), Add1 (+1.3), Daam2 (+1.4), Coro1b (+1.3) |
| --- | --- | --- |
| Microtubule | 4.05E-04 | Eml1 (+4.9), Kif21a (+1.9), Clip2 (+1.9), Ska2 (+1.6), Cep170  (+1.4), Rassf3 (+2.5), Eml4 (+1.4), Prc1 (+2.1), Tubg2  (+52.6), Rassf1 (+1.8), Kif23 (+2.6), Tpx2 (+1.9), Gtse1  (+2.0), Disc1 (+22.4), Tuba8 (+83.7), Ska3 (+2.6), Clasp2  (+1.5), Map1a (+3.9), Nckap5l (+2.8), Knstrn (+2.0), Map7  (+8.8), Map1b (+1.3), Tppp (+5.0), Dync1i1 (+1.6), Kif27  (+7.4), Dnm1 (+3.6), Shroom1 (+6.7), Kifc5b (+3.7), Spag5  (+1.9), Cep57 (+1.5), Aurka (+2.5), Map4 (+1.3), Kif22 (+1.9),  Kif11 (+1.4), Dynlt3 (+1.3), Rmdn2 (+2.1), Birc5 (+2.3),  Tubb6 (+1.8), Cep57l1 (+2.6), Dnah17 (+4.6), Camsap3  (+83.8), Nusap1 (+2.0), Ndrg1 (+3.5), Nek2 (+2.5), Kif20a  (+2.3), Kif4 (+1.8), Kif14 (+2.2) |
| GO:0000226~microtubule cytoskeleton organization | 4.17E-04 | Eml1 (+4.9), Aurka (+2.5), Bbs4 (+2.4), Tacc2 (+1.3), Tacc1  (+1.3), Prc1 (+2.1), Vamp4 (+1.4), Cnp (+2.0), Birc5 (+2.3),  Disc1 (+22.4), Tbce (+1.6), Tuba8 (+83.7), Clasp2 (+1.5),  Cul9 (+8.3), Map1a (+3.9), Obsl1 (+1.4), Map7 (+8.8), Map1b  (+1.3), Camsap3 (+83.8), Nusap1 (+2.0), Tacc3 (+2.2), Cul7  (+1.9), Gapdh (+1.5) |
| GO:0005874~microtubule | 6.84E-04 | Eml1 (+4.9), Sept9 (+1.3), Clip2 (+1.9), Rassf3 (+2.5), Kif23  (+2.6), Tubg2 (+52.6), Disc1 (+22.4), Ska3 (+2.6), Map1a  (+3.9), Dync1i1 (+1.6), Shroom1 (+6.7), Cep57 (+1.5), Aurka  (+2.5), Map4 (+1.3), Kif22 (+1.9), Kif11 (+1.4), Dynlt3 (+1.3),  Tubb6 (+1.8), Nusap1 (+2.0), Ndrg1 (+3.5), Bin1 (+2.1),  Kif21a (+1.9), Cep170 (+1.4), Ska2 (+1.6), Eml4 (+1.4),  Dpysl2 (+1.3), Prc1 (+2.1), Rassf1 (+1.8), Aspm (+1.6), Tpx2  (+1.9), Gtse1 (+2.0), Tuba8 (+83.7), Clasp2 (+1.5), Nckap5l  (+2.8), Knstrn (+2.0), Map1b (+1.3), Map7 (+8.8), Tppp  (+5.0), Kif27 (+7.4), Dnm1 (+3.6), Kifc5b (+3.7), Spag5  (+1.9), Map2k1 (+1.8), Rmdn2 (+2.1), Birc5 (+2.3), Cenpe  (+1.8), Gsk3b (+1.4), Cct5 (+1.3), Cep57l1 (+2.6), Dnah17  (+4.6), Camsap3 (+83.8), Nek2 (+2.5), Kif4 (+1.8), Kif14  (+2.2), Kif20a (+2.3) |
| GO:0008017~microtubule binding | 8.34E-04 | Eml1 (+4.9), Kif21a (+1.9), Clip2 (+1.9), Ska2 (+1.6), Eml4  (+1.4), Dpysl2 (+1.3), Prc1 (+2.1), Neil2 (+6.7), Kif23 (+2.6),  Clasp2 (+1.5), Map1a (+3.9), Map1b (+1.3), Tppp (+5.0),  Cryab (+7.7), Dync1i1 (+1.6), Dnm1 (+3.6), Kif27 (+7.4),  Spag5 (+1.9), Cep57 (+1.5), Gapdh (+1.5), Plk1 (+3.1), Nf1  (+1.3), Map4 (+1.3), Kif22 (+1.9), Kif11 (+1.4), Racgap1  (+2.6), Vapa (+1.8), Birc5 (+2.3), Cenpe (+1.8), Cep57l1  (+2.6), Soga2 (+2.1), Reep1 (+47.1), Camsap3 (+83.8),  Nusap1 (+2.0), Ndrg1 (+3.5), Kif20a (+2.3), Kif4 (+1.8), Kif14  (+2.2), Rab11a (+1.3) |
| GO:0030027~lamellipodium | 1.11E-03 | Arhgef6 (+5.3), Amotl1 (+1.3), Tesc (+1.6), Trpv2 (+52.4),  Ablim1 (+2.2), Pxn (+1.3), Dbn1 (+1.4), Vil1 (+9.7), Parvb |

|  |  | (+12.8), Klhl2 (+1.7), Snap25 (+7.2), Cdk5 (+1.3), Plekhg5  (+1.4), Twf2 (+1.8), Ctnnb1 (+1.6), Arhgef7 (+1.3), Tmod3  (+1.4), Plxnd1 (+1.6), Arap3 (+6.4), Podxl (+2.4), Mylk (+2.2),  Spata13 (+2.0), Myh10 (+20.6), Pld2 (+1.5), Palld (+1.8),  Pak1 (+2.6), Kitl (+1.5), Srgap2 (+1.3), Iqgap2 (+2.0), Tiam2  (+2.0), Rapgef3 (+6.0), Cttn (+1.4), Coro1b (+1.3) |
| --- | --- | --- |
| GO:0015629~actin cytoskeleton | 1.19E-03 | Sept9 (+1.3), Twf1 (+1.4), Coro2b (+6.2), Fyb (+8.0),  Arhgap32 (+1.3), Cit (+2.4), Pgm2 (+1.6), H1f0 (+2.3), Triobp  (+1.4), Ablim1 (+2.2), Kntc1 (+2.0), Rai14 (+1.9), Rara (+1.5),  Notch3 (+11.2), Dbn1 (+1.4), Smtn (+1.4), Klhl2 (+1.7),  Myoz2 (+91.0), Snap25 (+7.2), Dennd2a (+7.9), Adam17  (+1.3), Msra (+8.8), Pdlim7 (+1.5), Coro2a (+16.1), Coro6  (+4.0), Pou6f1 (+6.8), Ivns1abp (+1.8), Myh10 (+20.6), Maea  (+1.4), Palld (+1.8), Pdlim2 (+2.2), Anln (+2.0), Slc16a3  (+26.5), Baiap2l1 (+1.2), Baiap2 (+1.3), Aldoa (+1.4), Mtss1  (+1.9), Coro1b (+1.3) |
| GO:0008092~cytoskeletal protein binding | 4.88E-03 | Pick1 (+1.4), B4galt1 (+1.3), Des (+9.1), Pacsin1 (+24.4),  Map3k1 (+1.6), Aldoc (+1.5), Anxa2 (+1.4), Palld (+1.8),  Pacsin2 (+1.6), Capn1 (+1.6), Dbn1 (+1.4), Cryab (+7.7),  Tor1aip1 (+1.3), Aldoa (+1.4), Cdk5r1 (+4.1), Coro1b (+1.3),  Cdk5 (+1.3), Capn2 (+1.3) |
| GO:0051015~actin filament binding | 8.35E-03 | Pick1 (+1.4), Bin1 (+2.1), Coro2b (+6.2), Sptb (+2.6), Coro2a  (+16.1), Coro6 (+4.0), Myh14 (+15.9), Myo7a (+3.1), Triobp  (+1.4), Hip1 (+1.5), Gas7 (+8.3), Myh10 (+20.6), Syne3  (+1.5), Actn3 (+4.2), Myo18a (+1.3), Micall2 (+2.4), Adssl1  (+5.1), Dbn1 (+1.4), Iqgap2 (+2.0), Tpm1 (+1.3), Vil1 (+9.7),  Add1 (+1.3), Fmnl1 (+2.2), Shroom1 (+6.7), Ctnnal1 (+1.6),  Mtss1 (+1.9), Coro1b (+1.3) |
| mmu04510:Focal adhesion | 9.85E-03 | Ppp1cc (+1.4), Rap1b (+1.3), Myl12b (+1.4), Mylk4 (+136.2),  Pxn (+1.3), Vegfa (+7.5), Prkca (+1.9), Fn1 (+1.7), Parvb  (+12.8), Pdgfa (+2.6), Pdgfra (+5.6), Mapk3 (+1.5), Spp1  (+1.8), Col6a3 (+15.5), Ctnnb1 (+1.6), Itgb5 (+1.6), Akt3  (+6.7), Pik3cd (+3.9), Map2k1 (+1.8), Itga11 (+85.4), Shc1  (+1.5), Lamb1 (+1.4), Mylk (+2.2), Birc2 (+1.5), Itga5 (+1.9),  Gsk3b (+1.4), Pgf (+180.4), Actn3 (+4.2), Col3a1 (+8.2), Vwf  (+6.9), Ppp1cb (+1.3), Pak1 (+2.6), Ccnd2 (+2.8), Myl9  (+70.2), Cav1 (+2.7), Capn2 (+1.3), Col5a1 (+4.1) |
| Motor protein | 1.01E-02 | Kif21a (+1.9), Myh14 (+15.9), Myl6b (+2.6), Myh7b (+51.6),  Myo7a (+3.1), Kif23 (+2.6), Myo7b (+15.4), Myo18a (+1.3),  Myl12b (+1.4), Dync1i1 (+1.6), Kif27 (+7.4), Dnm1 (+3.6),  Kifc5b (+3.7), Kif22 (+1.9), Kif11 (+1.4), Dynlt3 (+1.3), Myo5b  (+3.6), Cenpe (+1.8), Myh10 (+20.6), Myl9 (+70.2), Dnah17  (+4.6), Myo1h (+2.7), Kif14 (+2.2), Kif4 (+1.8), Kif20a (+2.3) |
| GO:0005925~focal adhesion | 1.04E-02 | Pdlim1 (+8.7), Twf1 (+1.4), Pcbp2 (+1.4), Ppp1cc (+1.4),  Afap1 (+2.3), Anxa6 (+1.7), Pdcd6ip (+1.3), Mme (+4.6),  Kif23 (+2.6), Stard8 (+29.2), Parvb (+12.8), Hmga1 (+2.2),  Tspan9 (+1.7), Ppfibp1 (+2.6), Adam17 (+1.3), Arhgef7  (+1.3), Ctnnb1 (+1.6), Tns1 (+28.1), Kif22 (+1.9), Sh3kbp1  (+2.2), Dab2 (+1.6), Lims2 (+59.6), Bsg (+1.4), Sorbs3  (+1.6), Pdlim2 (+2.2), Pak1 (+2.6), Cttn (+1.4), Hspb1 (+2.1),  Cav1 (+2.7), Gna12 (+1.6), Capn2 (+1.3), Cd97 (+1.7),  Slc4a2 (+1.5), Efnb2 (+1.8), Triobp (+1.4), Pabpc1 (+1.8),  Clasp2 (+1.5), Pxn (+1.3), Pacsin2 (+1.6), Dixdc1 (+8.7),  Capn1 (+1.6), Mrc2 (+2.5), Cd44 (+1.4), Mapk3 (+1.5),  Ywhaz (+1.6), Pdlim7 (+1.5), Itgb5 (+1.6), Vim (+1.4), Capn5 |

|  |  | (+2.2), Fzd1 (+2.7), Map2k1 (+1.8), Itga11 (+85.4), Itga5  (+1.9), Palld (+1.8), Ppp1cb (+1.3), Slc9a3r2 (+1.4), Add1  (+1.3), Coro1b (+1.3) |
| --- | --- | --- |
| GO:0030175~filopodium | 1.33E-02 | Twf2 (+1.8), Twf1 (+1.4), Cd302 (+56.3), B4galt1 (+1.3),  Podxl (+2.4), Spata13 (+2.0), Def6 (+121.2), Palld (+1.8), Kitl  (+1.5), Dbn1 (+1.4), Iqgap2 (+2.0), Vil1 (+9.7), Tiam2 (+2.0),  Rapgef3 (+6.0), Snap25 (+7.2), Baiap2 (+1.3), Npcd (+1.6),  Cdk5 (+1.3) |
| GO:0098641~cadherin binding involved in cell-cell adhesion | 3.00E-02 | Arfip1 (+1.3), Sept9 (+1.3), Twf1 (+1.4), Pdlim1 (+8.7),  Ppme1 (+1.6), Ldha (+1.3), Sptbn2 (+4.6), Dab2ip (+1.5),  Anxa2 (+1.4), Gprc5a (+2.6), Krt18 (+1.3), Pacsin2 (+1.6),  Notch3 (+11.2), Ccnb2 (+2.2), Epcam (+4.9), Dbn1 (+1.4),  Stk24 (+1.4), Ppfibp1 (+2.6), Ywhaz (+1.6), Ubfd1 (+1.4),  Twf2 (+1.8), Ctnnb1 (+1.6), Puf60 (+1.3), Tmod3 (+1.4),  Vapa (+1.8), F11r (+1.3), Micall1 (+1.5), Lad1 (+13.9),  Ppp1r13l (+1.7), Bsg (+1.4), Eno1 (+1.5), Slc9a3r2 (+1.4),  C330027C09Rik (+2.4), Asap1 (+1.7), Anln (+2.0), Pfkp  (+1.9), Baiap2l1 (+1.2), Add1 (+1.3), Cttn (+1.4), Ndrg1  (+3.5), Baiap2 (+1.3), Aldoa (+1.4), Coro1b (+1.3), Tagln2  (+1.5) |
| GO:0005913~cell-cell adherens junction | 3.08E-02 | Arfip1 (+1.3), Fat2 (+10.5), Sept9 (+1.3), Twf1 (+1.4), Pdlim1  (+8.7), Ppme1 (+1.6), Ldha (+1.3), Sptbn2 (+4.6), Dab2ip  (+1.5), Anxa2 (+1.4), Gprc5a (+2.6), Krt18 (+1.3), Pacsin2  (+1.6), Notch3 (+11.2), Ccnb2 (+2.2), Dbn1 (+1.4), Stk24  (+1.4), Ppfibp1 (+2.6), Ywhaz (+1.6), Ubfd1 (+1.4), Twf2  (+1.8), Ctnnb1 (+1.6), Puf60 (+1.3), Tmod3 (+1.4), Vapa  (+1.8), F11r (+1.3), Micall1 (+1.5), Cdh3 (+3.1), Lad1 (+13.9),  Ppp1r13l (+1.7), Bsg (+1.4), Cadm1 (+1.5), Eno1 (+1.5),  Slc9a3r2 (+1.4), C330027C09Rik (+2.4), Dsc2 (+1.7), Asap1  (+1.7), Anln (+2.0), Pfkp (+1.9), Baiap2l1 (+1.2), Add1 (+1.3),  Cttn (+1.4), Ndrg1 (+3.5), Baiap2 (+1.3), Aldoa (+1.4),  Coro1b (+1.3), Tagln2 (+1.5) |

| **Metabolism** | **q-value** | **Gene symbols with fold change (FC)** |
| --- | --- | --- |
| mmu00010:Glycolysis / Gluconeogenesis#3 | 8.15E-06 | Tpi1 (+1.8), Pgam1 (+1.6), Aldh3b1 (+1.6), Aldoc (+1.5),  Ldha (+1.3), Hk2 (+1.9), Pgm2 (+1.6), Aldh2 (+1.3), Eno2  (+8.3), Galm (+15.6), Aldh3a1 (+1.6), Pfkm (+1.7), Aldh7a1  (+1.8), Pfkl (+1.5), Eno1 (+1.5), Gpi1 (+1.8), Eno3 (+1.7),  Pfkp (+1.9), Pck2 (+1.4), Aldoa (+1.4), Hk1 (+1.5), Gapdh  (+1.5) |
| mmu05230:Central carbon metabolism in cancer | 3.59E-05 | Mtor (+1.3), Pgam1 (+1.6), Pdk1 (+2.2), G6pdx (+1.3), Hk2  (+1.9), Pik3cd (+3.9), Map2k1 (+1.8), Akt3 (+6.7), Pfkm  (+1.7), Slc7a5 (+1.9), Kit (+43.4), Pfkl (+1.5), Slc2a1 (+1.7),  Fgfr1 (+1.6), Pfkp (+1.9), Slc16a3 (+26.5), Gls2 (+3.2), Fgfr3  (+3.6), Pdgfra (+5.6), Hk1 (+1.5), Mapk3 (+1.5) |
| Glycolysis | 3.87E-05 | Pgam1 (+1.6), Tpi1 (+1.8), Aldoc (+1.5), Hk2 (+1.9), Eno2  (+8.3), Pfkm (+1.7), Pfkl (+1.5), Eno1 (+1.5), Gpi1 (+1.8),  Eno3 (+1.7), Pfkp (+1.9), Aldoa (+1.4), Hk1 (+1.5), Gapdh  (+1.5) |
| mmu00480:Glutathione metabolism | 4.02E-05 | Mgst2 (+13.6), Gsta3 (+4.9), G6pdx (+1.3), Gpx8 (+2.1),  Gstm6 (+816.4), Ggt5 (+5.2), Ggt6 (+3.6), Rrm2b (+1.4),  Gstm1 (+6.9), Gstt1 (+5.0), Gstm7 (+19.2), Gstt3 (+2.4), |

|  |  | Gstk1 (+38.2), Anpep (+11.7), Gstm4 (+4.7), Gstm2 (+2.6),  Oplah (+1.6), Gsta2 (+5.7), Rrm1 (+1.5) |
| --- | --- | --- |
| GO:0006096~glycolytic process | 1.51E-04 | Pgam1 (+1.6), Tpi1 (+1.8), Aldoc (+1.5), Hk2 (+1.9), Eno2  (+8.3), Pfkm (+1.7), Pfkl (+1.5), Eno1 (+1.5), Gpi1 (+1.8),  Eno3 (+1.7), Pfkp (+1.9), Aldoa (+1.4), Hk1 (+1.5), Gapdh  (+1.5) |
| mmu00052:Galactose metabolism | 4.80E-04 | B4galt1 (+1.3), Glb1 (+1.4), Hk2 (+1.9), Pgm2 (+1.6), Akr1b8  (+3.7), Galm (+15.6), Pfkm (+1.7), Pfkl (+1.5), Akr1b3 (+3.6),  Pfkp (+1.9), Hk1 (+1.5), Akr1b7 (+77.8), Akr1b10 (+2.2) |
| GO:0004364~glutathione transferase activity | 5.41E-04 | Mgst2 (+13.6), Gstm1 (+6.9), Gstm7 (+19.2), Gstt1 (+5.0),  Gstt3 (+2.4), Gsta3 (+4.9), Gstz1 (+1.8), Gstm6 (+816.4),  BC021614 (+166.2), Gstk1 (+38.2), Gstm4 (+4.7), Gstm2  (+2.6), Gsta2 (+5.7) |
| GO:0008152~metabolic process | 7.61E-04 | Eci2 (+1.5), Tpi1 (+1.8), Naga (+1.4), Fuca1 (+1.3), Gstz1  (+1.8), Mcat (+2.0), Nt5c3 (+1.4), Pfkm (+1.7), Gstm7  (+19.2), Sulf2 (+2.1), Acy3 (+1.6), Scp2 (+1.3), Enpp1 (+2.2),  Pgam1 (+1.6), Gsta3 (+4.9), Aldh3b1 (+1.6), Hk2 (+1.9),  Hadha (+1.3), Myo5b (+3.6), Aldh3a1 (+1.6), Aldh7a1 (+1.8),  Gstm1 (+6.9), Pak1 (+2.6), Aldh1l2 (+8.8), Enpp5 (+2.0),  Bcat1 (+3.0), Nadk2 (+2.6), Rrm1 (+1.5), Hk1 (+1.5), Gyltl1b  (+4.1), Agpat2 (+2.1), Aco2 (+1.3), Xrcc6 (+1.8), Acad10  (+1.9), Nt5c2 (+1.6), Tsta3 (+1.7), Neil2 (+6.7), Acaa2 (+1.4),  Aldh5a1 (+4.0), Aldh2 (+1.3), Pla2g6 (+1.4), Camk1 (+1.6),  Aldh1a2 (+8.3), Gys1 (+1.9), Alppl2 (+3.9), Echs1 (+1.5),  Gstm2 (+2.6), Arsa (+1.5), Gsta2 (+5.7), Lpcat2 (+3.5), Alpl  (+126.7), Acacb (+3.8), Slc27a1 (+3.2), Papss2 (+14.2),  Isoc2b (+2.4), Acsbg1 (+4.6), Gpam (+1.9), Hyal1 (+2.2),  Glb1 (+1.4), Gstm6 (+816.4), Acadsb (+1.4), Mthfd2 (+2.7),  Pfkl (+1.5), Arsb (+1.3), Aldh18a1 (+1.6), Aasdh (+1.9),  BC021614 (+166.2), Pfkp (+1.9), Ugdh (+1.7), Alad (+1.7),  Grhpr (+2.0), Hadhb (+1.6), Enpp2 (+4.9), Hexdc (+1.4) |
| mmu01100:Metabolic pathways | 3.87E-03 | Mthfs (+4.0), Ndufa6 (+1.5), Tpi1 (+1.8), Ugcg (+1.6), Aox4  (+10.7), Aldoc (+1.5), Mcat (+2.0), Pgm2 (+1.6), Glce (+2.1),  Nt5c3 (+1.4), Pfkm (+1.7), Gatm (+131.7), Gbe1 (+3.1),  Nampt (+1.5), Plcb4 (+1.9), Cyp2c44 (+28.5), Adssl1 (+5.1),  Cox17 (+1.5), Enpp1 (+2.2), Gfpt1 (+1.3), Khk (+2.6), Oat  (+1.6), Extl1 (+3.9), Ptdss2 (+1.7), Bcat1 (+3.0), Etnk2  (+11.6), Nadk2 (+2.6), Prim1 (+2.4), Nos1 (+63.9), St3gal5  (+2.0), Polr3h (+1.6), Aco2 (+1.3), Nt5c2 (+1.6), Tsta3 (+1.7),  Ppox (+1.8), Tk1 (+2.6), Pycrl (+1.7), Akr1b8 (+3.7), Aldh5a1  (+4.0), Pla2g6 (+1.4), Naprt1 (+4.6), Ndufb4 (+3.1), Aldh1a2  (+8.3), Nt5e (+5.6), Cyp27a1 (+9.9), Csad (+1.4), Pnliprp1  (+1.8), Gls2 (+3.2), Alpl (+126.7), Acacb (+3.8), Ass1 (+5.6),  Akr1b7 (+77.8), Acsbg1 (+4.6), P4ha2 (+2.1), Gpam (+1.9),  Pold3 (+1.5), Glb1 (+1.4), Mthfd2 (+2.7), Acadsb (+1.4), Tst  (+2.8), Atp6v1c2 (+9.8), Aox1 (+1.8), Bcmo1 (+70.9), Lipt2  (+2.5), Gfpt2 (+1.8), Arsb (+1.3), Pld2 (+1.5), Pnp2 (+118.5),  Nos2 (+12.7), Gpi1 (+1.8), Akr1b3 (+3.6), Ugdh (+1.7), Grhpr  (+2.0), P4ha1 (+2.2), Hadhb (+1.6), Ak1 (+1.3), Cda (+7.7),  Cpox (+1.8), G6pdx (+1.3), Gstz1 (+1.8), B4galt6 (+1.5), Gpt  (+10.8), Pcyt1b (+2.3), A4galt (+2.8), Eno2 (+8.3), Galm  (+15.6), Nmnat2 (+9.7), Glul (+1.4), Gmppa (+1.6), Polr2f  (+1.3), Plch1 (+2.1), St3gal2 (+1.3), Pip5k1b (+3.6), Scp2  (+1.3), Pon3 (+3.6), Gapdh (+1.5), Ak7 (+8.3), Pgam1 (+1.6),  Atp6v1c1 (+1.5), Aldh3b1 (+1.6), Cyp4f14 (+16.3), Pold4 |

|  |  | (+1.5), Hk2 (+1.9), Ndufa4l2 (+221.2), Hadha (+1.3), Ggt6  (+3.6), Aldh3a1 (+1.6), Aldh7a1 (+1.8), Rrm2b (+1.4), Ptgds  (+4.7), Cox6b2 (+17.3), Eno1 (+1.5), Uros (+2.0), Eno3  (+1.7), Extl3 (+1.4), Ext1 (+1.3), Anpep (+11.7), Ndufb9  (+1.5), Abat (+40.6), Rrm1 (+1.5), Hk1 (+1.5), Agpat2 (+2.1),  Degs1 (+1.3), B4galt1 (+1.3), Ogdhl (+201.2), Ldha (+1.3),  Aldh2 (+1.3), Hykk (+3.4), Acaa2 (+1.4), Hdc (+20.4), Alppl2  (+3.9), Pts (+1.7), Pla2g7 (+12.1), Amacr (+1.8), Echs1  (+1.5), B3gnt3 (+2.5), Pck2 (+1.4), Lpcat2 (+3.5), B4galnt1  (+2.1), Ada (+7.8), Papss2 (+14.2), Hyal1 (+2.2), Mpst  (+1.6), Ggt5 (+5.2), P4ha3 (+22.9), Hpse (+4.9), Galnt18  (+11.2), Cmas (+1.4), Pfkl (+1.5), Nags (+22.1), Aldh18a1  (+1.6), Galnt12 (+3.1), Cmbl (+33.7), Alg10b (+1.3), Pfkp  (+1.9), Alad (+1.7), Fam213b (+4.0), Man1c1 (+3.4), Aldoa  (+1.4), Akr1b10 (+2.2) |
| --- | --- | --- |
| GO:0006749~glutathione metabolic process | 4.15E-03 | Gsta3 (+4.9), G6pdx (+1.3), Gstz1 (+1.8), Ggt5 (+5.2), Ggt6  (+3.6), Aldh5a1 (+4.0), Gstt1 (+5.0), Gstm7 (+19.2), Gstm1  (+6.9), Gstt3 (+2.4), Gstk1 (+38.2), Gstm4 (+4.7), Oplah  (+1.6), Gstm2 (+2.6), Gsta2 (+5.7) |
| IPR004046:Glutathione S- transferase, C-terminal | 4.48E-03 | Gstm1 (+6.9), Gstm7 (+19.2), Gstt1 (+5.0), Gstt3 (+2.4),  Gsta3 (+4.9), Gstz1 (+1.8), Gstm6 (+816.4), BC021614  (+166.2), Gstm4 (+4.7), Gstm2 (+2.6), Gsta2 (+5.7) |
| GO:0005739~mitochondrion | 5.94E-03 | Mcat (+2.0), Nt5c3 (+1.4), Efhd1 (+194.2), Disc1 (+22.4),  Higd1a (+1.5), Nit1 (+1.8), Tatdn3 (+2.3), Ago2 (+1.6),  Rmnd1 (+1.6), Vdac1 (+1.4), Esr1 (+1.7), Mettl20 (+1.8),  Pdk4 (+3.7), Myh10 (+20.6), Oat (+1.6), Aldh1l2 (+8.8),  Bcat1 (+3.0), Hif3a (+4.4), Nadk2 (+2.6), Cav1 (+2.7), Nos1  (+63.9), Slc25a30 (+1.7), Aldh5a1 (+4.0), Fam73a (+2.3),  Abcd1 (+2.1), Bok (+1.5), Cyp27a1 (+9.9), Slc25a24 (+1.3),  Gls2 (+3.2), Acacb (+3.8), Ywhaz (+1.6), Mapk3 (+1.5),  As3mt (+22.0), Msra (+8.8), Isoc2b (+2.4), Pycard (+4.3),  Rab11fip5 (+1.7), Acbd3 (+1.3), Tst (+2.8), Map2k1 (+1.8),  Parp1 (+1.6), Tfrc (+2.1), Bcl2l2 (+1.3), Arsb (+1.3), Sccpdh  (+1.6), Hadhb (+1.6), 1110007C09Rik (+2.1), Lonp1 (+1.7),  Ppp1cc (+1.4), Cpox (+1.8), Dcakd (+1.4), Anxa6 (+1.7),  Hivep1 (+1.3), Ripk3 (+44.1), Ddah2 (+1.6), Plscr3 (+1.5),  Rab3d (+1.5), Zfp13 (+2.3), Lyrm5 (+1.8), Ccdc58 (+2.3),  Rsad2 (+8.9), Hk2 (+1.9), Slc25a13 (+1.4), Hadha (+1.3),  Tspo (+1.5), Aldh7a1 (+1.8), Clybl (+2.3), D2hgdh (+1.7),  Uros (+2.0), Hspa2 (+1.8), Ndufb9 (+1.5), Abat (+40.6),  Degs1 (+1.3), Ogdhl (+201.2), Prelid1 (+1.5), Dpysl2 (+1.3),  Ociad2 (+3.4), Hebp2 (+18.1), Ociad1 (+1.3), Myg1 (+1.4),  Ddit4 (+2.3), Sp140 (+4.8), Rnasel (+4.7), Rai14 (+1.9),  Amacr (+1.8), Elk3 (+1.6), Echs1 (+1.5), Xrcc3 (+5.1),  Slc27a1 (+3.2), Mpst (+1.6), Nags (+22.1), Aldh18a1 (+1.6),  Gpd1 (+7.3), Ldhd (+2.3), Adck5 (+1.4), Reep1 (+47.1),  Aldoa (+1.4), Ucp2 (+2.4), Mthfs (+4.0), Ndufa6 (+1.5),  Smdt1 (+1.5), Aldoc (+1.5), Acot13 (+1.4), Cyb5r3 (+1.5),  Oas1a (+16.2), Gatm (+131.7), Ap2m1 (+1.3), Cox17 (+1.5),  Malsu1 (+1.4), Cox14 (+1.7), Ppp3ca (+1.3), Shc1 (+1.5),  Maff (+1.5), Oxr1 (+1.9), Slc25a45 (+1.7), Ssbp1 (+1.8),  Hebp1 (+4.1), Aco2 (+1.3), Ppox (+1.8), Dact2 (+17.1),  Pla2g6 (+1.4), Ndufb4 (+3.1), Bnip3 (+8.4), Rab24 (+1.6),  Prkcd (+1.5), Vamp1 (+2.0), Ass1 (+5.6), Akr1b7 (+77.8),  Cerk (+2.4), Gm2a (+2.5), Gpam (+1.9), Etfa (+1.5), Pold3 |

|  |  | (+1.5), Acadsb (+1.4), Mthfd2 (+2.7), Rmdn2 (+2.1), Tert  (+1.6), Star (+1.8), Lipt2 (+2.5), Immt (+1.3), Hrsp12 (+2.2),  Gsk3b (+1.4), Guf1 (+1.5), P4ha1 (+2.2), Fastk (+1.7), Eci2  (+1.5), Maats1 (+8.5), Gstz1 (+1.8), Bphl (+2.0), Pmaip1  (+2.1), Glul (+1.4), Cpt1b (+3.8), Mtch1 (+1.4), Scp2 (+1.3),  Slc22a4 (+2.4), Gapdh (+1.5), Fam162a (+1.9), Pdk1 (+2.2),  Serhl (+1.5), Slc25a11 (+1.3), Bckdk (+1.4), Bsg (+1.4), Lrp5  (+6.1), Rrm2b (+1.4), Cox6b2 (+17.3), Slc25a17 (+1.5), Hk1  (+1.5), Tap1 (+2.0), Adck3 (+2.3), Acad10 (+1.9), Ldha  (+1.3), Pebp1 (+1.3), Arl2 (+1.4), Cdk1 (+1.8), Aldh2 (+1.3),  Acaa2 (+1.4), Tmem173 (+1.7), Ccdc90b (+2.6), Pts (+1.7),  Thra (+1.6), Prkca (+1.9), Capn1 (+1.6), Pck2 (+1.4), Bco2  (+9.2), Cryab (+7.7), Mtor (+1.3), Pick1 (+1.4), Phyh (+1.4),  Adhfe1 (+3.5), Sfxn4 (+2.8), Palld (+1.8), Lrrc24 (+32.1),  Kank2 (+1.7), Dmpk (+8.7), Gstk1 (+38.2), Gprc5c (+2.5),  Fdx1 (+1.6), Prelid2 (+20.4), Akr1b10 (+2.2), Rab11a (+1.3) |
| --- | --- | --- |
| IPR004045:Glutathione S- transferase, N-terminal | 8.26E-03 | Gstm1 (+6.9), Gstm7 (+19.2), Gstt1 (+5.0), Gstt3 (+2.4),  Gsta3 (+4.9), Gstz1 (+1.8), Gstm6 (+816.4), BC021614  (+166.2), Gstm4 (+4.7), Gstm2 (+2.6), Gsta2 (+5.7) |
| GO:0005975~carbohydrate metabolic process | 3.73E-02 | Stbd1 (+3.4), B4galt1 (+1.3), Naga (+1.4), Fuca1 (+1.3),  G6pdx (+1.3), B4galt6 (+1.5), Ldha (+1.3), Pgm2 (+1.6), Siae  (+1.7), Epm2a (+3.0), Galm (+15.6), Gbe1 (+3.1), Lctl (+3.1),  Ppp1r3c (+21.7), Chid1 (+1.5), Gapdh (+1.5), Gfpt1 (+1.3),  Pdk1 (+2.2), Ppp1r3b (+3.3), Khk (+2.6), Glb1 (+1.4), Hyal1  (+2.2), Hk2 (+1.9), Hpse (+4.9), Pdk4 (+3.7), Gfpt2 (+1.8),  Gsk3b (+1.4), Pfkl (+1.5), Ppp1cb (+1.3), Gpi1 (+1.8), Gpd1  (+7.3), Ugdh (+1.7), Fggy (+5.5), Hk1 (+1.5), Hexdc (+1.4) |

| **Down-regulation Lipid metabolism** | **q-value** | **Gene symbols with fold change (FC)** |
| --- | --- | --- |
| GO:0006695~cholesterol biosynthetic process | 2.85E-12 | Fdps (-4.2), Hsd17b7 (-4.0), Nsdhl (-4.1), Cyp51 (-2.4), Mvd  (-4.0), Idi1 (-3.3), Pmvk (-2.1), Cftr (-2.8), Hmgcs1 (-2.9),  Insig1 (-3.0), Dhcr24 (-5.1), Fdft1 (-2.1), Mvk (-2.2) |
| GO:0016126~sterol biosynthetic process | 1.53E-11 | Pmvk (-2.1), Hmgcs1 (-2.9), Insig1 (-3.0), Fdps (-4.2), Dhcr24  (-5.1), Nsdhl (-4.1), Fdft1 (-2.1), Sc4mol (-3.2), Cyp51 (-2.4),  Sqle (-2.9), Mvk (-2.2), Mvd (-4.0) |
| Sterol biosynthesis | 1.37E-10 | Pmvk (-2.1), Hmgcs1 (-2.9), Fdps (-4.2), Dhcr24 (-5.1), Nsdhl  (-4.1), Fdft1 (-2.1), Sc4mol (-3.2), Cyp51 (-2.4), Mvk (-2.2),  Idi1 (-3.3), Mvd (-4.0) |
| Cholesterol biosynthesis | 2.33E-10 | Pmvk (-2.1), Hmgcs1 (-2.9), Fdps (-4.2), Dhcr24 (-5.1), Nsdhl  (-4.1), Fdft1 (-2.1), Cyp51 (-2.4), Mvk (-2.2), Idi1 (-3.3), Mvd  (-4.0) |
| Steroid biosynthesis | 5.34E-10 | Pmvk (-2.1), Hmgcs1 (-2.9), Fdps (-4.2), Dhcr24 (-5.1),  Hsd17b7 (-4.0), Nsdhl (-4.1), Fdft1 (-2.1), Sc4mol (-3.2),  Cyp51 (-2.4), Mvk (-2.2), Idi1 (-3.3), Mvd (-4.0) |
| Sterol metabolism | 7.25E-10 | Fdps (-4.2), Nsdhl (-4.1), Cyp51 (-2.4), Sorl1 (-2.1), Mvd (-  4.0), Idi1 (-3.3), Pmvk (-2.1), Hmgcs1 (-2.9), Insig1 (-3.0),  Ldlr (-2.5), Dhcr24 (-5.1), Fdft1 (-2.1), Sc4mol (-3.2), Mvk (-  2.2) |
| Cholesterol metabolism | 2.32E-09 | Fdps (-4.2), Nsdhl (-4.1), Cyp51 (-2.4), Sorl1 (-2.1), Mvd (-  4.0), Idi1 (-3.3), Pmvk (-2.1), Hmgcs1 (-2.9), Insig1 (-3.0),  Ldlr (-2.5), Dhcr24 (-5.1), Fdft1 (-2.1), Mvk (-2.2) |

| Steroid metabolism | 5.64E-09 | Fdps (-4.2), Nsdhl (-4.1), Cyp51 (-2.4), Sorl1 (-2.1), Mvd (-  4.0), Idi1 (-3.3), Pmvk (-2.1), Hmgcs1 (-2.9), Insig1 (-3.0),  Ldlr (-2.5), Dhcr24 (-5.1), Fdft1 (-2.1), Sc4mol (-3.2), Mvk (-  2.2) |
| --- | --- | --- |
| GO:0006629~lipid metabolic process | 2.67E-05 | Fabp5 (-2.8), Gdpd1 (-2.2), Fdps (-4.2), Acsl1 (-3.4),  Hsd17b7 (-4.0), Cyp51 (-2.4), Tnxb (-4.1), Mvd (-4.0), Insig1  (-3.0), Pnliprp2 (-6.7), Acaca (-2.4), Sc4mol (-3.2), Ptgs2 (-  3.8), Ppap2a (-2.2), Nsdhl (-4.1), Fasn (-2.4), Sorl1 (-2.1),  Fads2 (-2.4), Pmvk (-2.1), Hmgcs1 (-2.9), Gdpd2 (-2.1), Ldlr  (-2.5), Dhcr24 (-5.1), Fdft1 (-2.1), Sgms2 (-2.2), Mvk (-2.2) |
| mmu00100:Steroid biosynthesis | 3.31E-05 | Dhcr24 (-5.1), Hsd17b7 (-4.0), Nsdhl (-4.1), Fdft1 (-2.1),  Sc4mol (-3.2), Cyp51 (-2.4), Sqle (-2.9) |
| Lipid metabolism | 3.13E-04 | Fdps (-4.2), Acsl1 (-3.4), Hsd17b7 (-4.0), Nsdhl (-4.1), Fasn  (-2.4), Cyp51 (-2.4), Sorl1 (-2.1), Mvd (-4.0), Idi1 (-3.3), Pmvk  (-2.1), Fads2 (-2.4), Hmgcs1 (-2.9), Insig1 (-3.0), Ldlr (-2.5),  Pnliprp2 (-6.7), Dhcr24 (-5.1), Acaca (-2.4), Fdft1 (-2.1),  Sgms2 (-2.2), Sc4mol (-3.2), Mvk (-2.2), Ptgs2 (-3.8) |

The associated genes in the pathways with corresponding q-values are shown.

The fold changes (ID8-P2 vs. ID8-P0) are enclosed in the bracket.
