# Supplementary material for: Changes in mRNA/protein expression and signaling pathways in in vivo passaged mouse ovarian cancer cells
Source: PLoS One. 2018 Jun 21;13(6):e0197404. doi: 10.1371/journal.pone.0197404 (PMC6013233; doi:10.1371/journal.pone.0197404)
Supplement: S3 Table — The associated genes in the pathways with corresponding q-values are shown. The fold changes (ID8-P2 vs. ID8-P0) are enclosed in the bracket. (DOCX) [file pone.0197404.s004.docx]

# S3 Table DEGs up-regulated in ID8-PW2 associated with pH pathway.

| **SMART** | **q-value** | **Gene symbols with FC** |
| --- | --- | --- |
| SM00233:PH | 1.19E-05 | Afap1 (+2.3), Cit (+2.4), Arhgef28 (+5.9), Cyth4 (+22.7), Rtkn (+1.8),  Sptbn2 (+4.6), Dab2ip (+1.5), Cnksr1 (+233.9), Ngef (+8.7), Arhgap9  (+4.2), Osbp2 (+3.6), Arap2 (+1.8), Plch1 (+2.1), Net1 (+1.9), Rasal1  (+2.1), Arhgef40 (+4.2), Plekhg5 (+1.4), Arhgef7 (+1.3), Sptb (+2.6),  Plekha2 (+1.5), Arap3 (+6.4), Rasa1 (+1.5), Phldb1 (+1.6), Afap1l1  (+84.1), Plekhg4 (+7.0), Psd4 (+8.1), Syngap1 (+1.7), Plcl1 (+5.7),  Plekho1 (+4.6), Arhgef6 (+5.3), Triobp (+1.4), Plekhg6 (+5.2), Arap1  (+3.1), Dnm1 (+3.6), Rtkn2 (+7.2), Dock10 (+17.4), Gab2 (+3.2),  Arhgef19 (+1.8), Akt3 (+6.7), Plekha6 (+1.7), Plekhn1 (+2.2), Prkd3  (+1.3), Spata13 (+2.0), Def6 (+121.2), Pld2 (+1.5), Acap1 (+10.8),  Anln (+2.0), Asap1 (+1.7), Sbf1 (+1.3), Osbpl9 (+1.3), Fam109b  (+143.1), Tiam2 (+2.0), Rasal2 (+1.7), Arhgef3 (+7.6) |

The associated genes in the pathways with corresponding q-values are shown.

The fold changes (ID8-P2 vs. ID8-P0) are enclosed in the bracket.
